# Aging is associated with a decline in Atg9b‐mediated autophagosome formation and appearance of enlarged mitochondria in the heart

**DOI:** 10.1111/acel.13187

**Published:** 2020-07-06

**Authors:** Wenjing Liang, Alexandra G. Moyzis, Mark A. Lampert, Rachel Y. Diao, Rita H. Najor, Åsa B. Gustafsson

**Affiliations:** ^1^ Department of Pharmacology Skaggs School of Pharmacy and Pharmaceutical Sciences University of California, San Diego La Jolla California USA

**Keywords:** aging, Atg9, autophagy, heart, mitochondria, mitophagy, Parkin

## Abstract

Advancing age is a major risk factor for developing heart disease, and the biological processes contributing to aging are currently under intense investigation. Autophagy is an important cellular quality control mechanism that is reduced in tissues with age but the molecular mechanisms underlying the age‐associated defects in autophagy remain poorly characterized. Here, we have investigated how the autophagic process is altered in aged mouse hearts. We report that autophagic activity is reduced in aged hearts due to a reduction in autophagosome formation. Gene expression profile analysis to evaluate changes in autophagy regulators uncovered a reduction in Atg9b transcript and protein levels. Atg9 proteins are critical in delivering membrane to the growing autophagosome, and siRNA knockdown of Atg9b in cells confirmed a reduction in autophagosome formation. Autophagy is also the main pathway involved in eliminating dysfunctional mitochondria via a process known as mitophagy. The E3 ubiquitin ligase Parkin plays a key role in labeling mitochondria for mitophagy. We also found increased levels of Parkin‐positive mitochondria in the aged hearts, an indication that they have been labeled for mitophagy. In contrast, Nrf1, a major transcriptional regulator of mitochondrial biogenesis, was significantly reduced in aged hearts. Additionally, our data showed reduced Drp1‐mediated mitochondrial fission and formation of enlarged mitochondria in the aged heart. Overall, our findings suggest that cardiac aging is associated with reduced autophagosome number, decreased mitochondrial turnover, and formation of megamitochondria.

## Graphical abstract

Here, we report that autophagic activity is reduced in aged hearts due to decreased expression of the autophagy‐related protein Atg9b. This protein is involved in delivering membrane to form and expand the autophagosome. Our findings also demonstrate that there is an imbalance in the labelling and degradation steps in the aged myocardium due to reduced formation of autophagosomes. The decline in mitochondrial clearance also coincides with formation of megamitochondria in aged hearts.

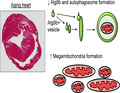

## 1 INTRODUCTION

Aging is associated with a gradual decline in tissue functions, which can lead to age‐related disorders. Advancing age is considered a major risk factor for heart disease, and intrinsic alterations in aging cardiac myocytes likely contribute to the underlying pathogenesis (Dai & Rabinovitch, 2009). Autophagy is an important cellular quality control mechanism, and defects in this process lead to reduced removal of cytotoxic protein aggregates and damaged organelles. Several studies have demonstrated that enhancing autophagy increases life span in various organisms ranging from worms to mice (Eisenberg et al., 2016; Fernandez et al., 2018; Nakamura et al., 2019; Pyo et al., 2013; Simonsen et al., 2008; Toth et al., 2008). There is also evidence that autophagy decreases with age in tissues, including the heart (Inuzuka et al., 2009; Nakamura et al., 2019; Ren et al., 2017; Taneike et al., 2010; Zhou et al., 2017). However, the mechanisms underlying the age‐related decline in autophagy remain unclear.

Autophagy is also the main pathway involved in eliminating mitochondria in a process known as mitophagy. This is a highly regulated process involving two coordinated steps to ensure the selective removal of dysfunctional mitochondria. The first step involves activation of the E3 ubiquitin ligase Parkin which is responsible for labeling damaged mitochondria for degradation (Narendra, Tanaka, Suen, & Youle, 2008). Parkin is cytosolic under basal conditions but is recruited to dysfunctional mitochondria by PINK1, where it then proceeds to ubiquitinate various proteins in the outer membrane (Narendra et al., 2008; Suen, Narendra, Tanaka, Manfredi, & Youle, 2010). The ubiquitinated proteins serve as a signal for an autophagosome to sequester the mitochondrion (Geisler et al., 2010). The second event in mitophagy is the concurrent formation of autophagosomes, which are responsible for engulfing and delivering the ubiquitinated mitochondria to lysosomes for degradation. The importance of Parkin in clearing damaged mitochondria in myocytes during acute stress is well established (Hoshino et al., 2013; Kubli, Quinsay, & Gustafsson, 2013). Also, overexpression of Parkin leads to increased life span in *Drosophila* (Rana, Rera, & Walker, 2013). While Parkin^−/−^ mice accumulate abnormal mitochondria in the heart with age (Hoshino et al., 2013; Kubli et al., 2013), overexpression of Parkin preserves mitochondrial function in aging mouse hearts (Hoshino et al., 2013). However, cardiac Parkin overexpression or systemic deficiency has little effect on the accelerated cardiac aging phenotype in mtDNA mutator mice, suggesting a limited role for Parkin‐mediated mitophagy in this aging mouse model (Woodall et al., 2019). These studies indicate a role for Parkin preventing the aging process, but whether Parkin‐mediated mitophagy is altered in the aged heart is currently unclear.

To date, most mechanistic studies on autophagy and mitophagy in aging have been restricted to lower organisms and how these processes are affected at the molecular level are still lacking in mammalian systems. In this study, we demonstrate that aging is associated with decreased expression of the autophagy‐related protein Atg9b which correlates with reduced formation of autophagosomes in the aged heart. Our data also show that there is an increase in mitochondria that have been labeled for mitophagy, indicating a potential imbalance in the mitophagy process in aged hearts. Finally, we found that decreased autophagy also coincided with reduced Drp1‐mediated fission and formation of enlarged mitochondria.

## 2 RESULTS

### 2.1 Characterization of aged mice

**FIGURE 1:**
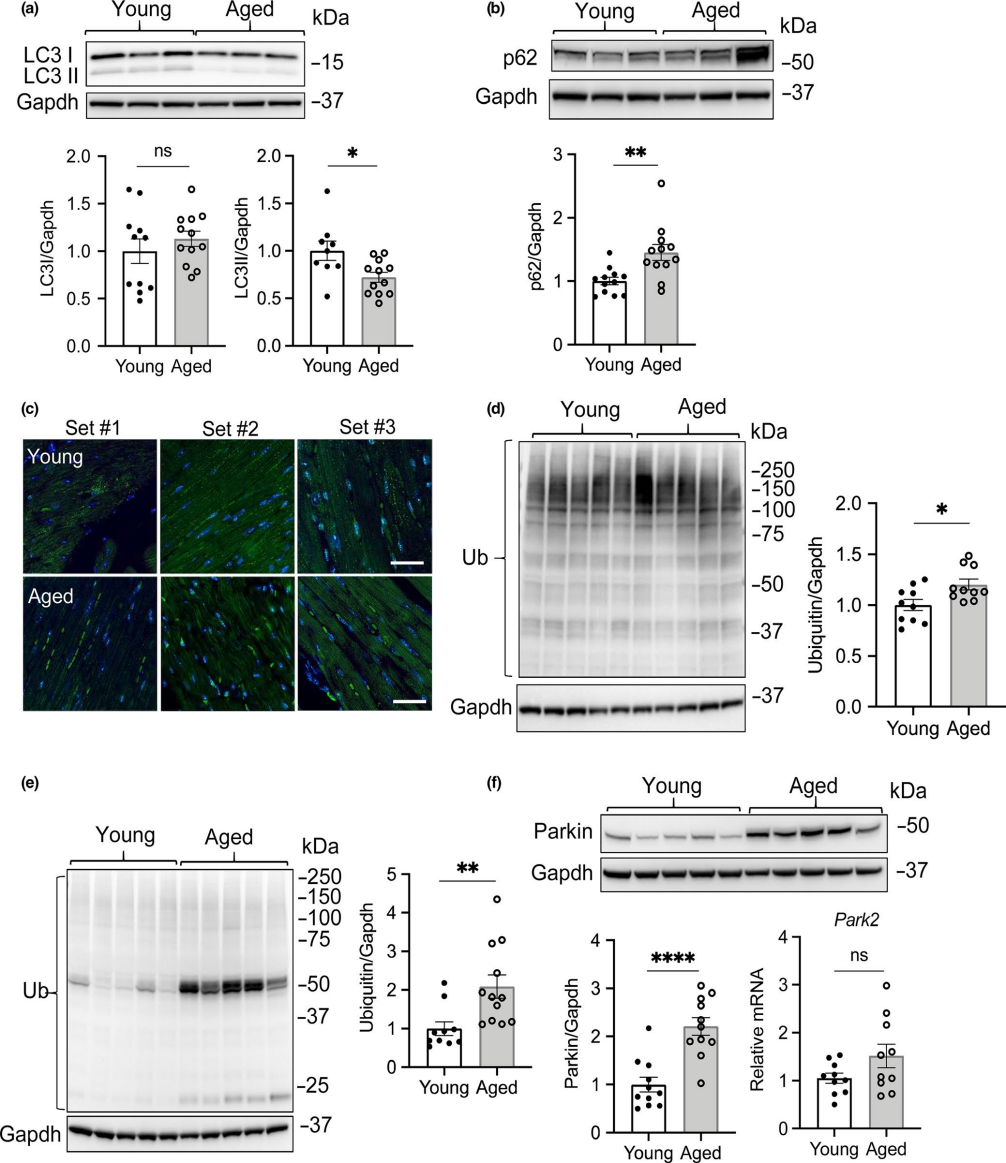
Assessment of general autophagy in hearts from young and aged mice. (a) Representative Western blot and quantitation of LC3 levels from heart lysates of young and aged mice (*n* = 9–12). (b) Representative Western blot and quantitation of p62 levels in young and aged hearts (*n* = 11–12). (c) Representative images for LC3 immunostaining in heart sections (*n* = 3 for each). (d) Representative Western blot and quantitation of protein ubiquitination in young and aged hearts using a polyclonal ubiquitin antibody (*n* = 10). (e) Representative Western blot and quantitation of protein ubiquitination in young and aged hearts using a monoclonal ubiquitin antibody (*n* = 10–12). (f) Representative Western blot and quantitation of Parkin protein and transcript levels in young and aged hearts (*n* = 10–12). Scale bars are 20 μm. Data represent the mean ± SEM (*p < 0.05, **p < 0.01, ****p < 0.0001, ns = not significant)

To examine the effect of aging on the heart, we evaluated cardiac structure and function in 4 (young)‐ and 24 (aged)‐month‐old male mice. We found no significant differences in ejection fraction (EF) or fractional shortening (FS), left ventricular internal end‐diastolic and systolic dimensions (LVID;d and LVID;s) between young and old mice (Figure S1a). However, aging is associated with diastolic dysfunction and using the pulse Doppler wave mode to assess the E/A ratio revealed a significant decrease in E/A ratio in aged hearts (Figure S1b). We also found that aged mice had a significant increase in heart weight/body weight (HW/BW) and heart weight/tibia length (HW/TL) ratios compared to young mice (Figure S1c). Additionally, we found significantly elevated levels of β myosin heavy chain (*Myh7*), while transcript levels of inflammatory markers, interleukin‐6 *(IL*‐*6)* and tumor necrosis factor alpha (*Tnfα*), trended higher in the aged hearts (Figure 1d). Haematoxylin and eosin (H&E) staining showed similar structure in young and aged heart sections, while Masson's trichrome staining revealed increased perivascular, but not interstitial, fibrosis in the aged hearts (Figure S1e). Despite the increase in perivascular fibrosis, transforming growth factor beta (*Tgfβ)*, type I collagen (*Col1)*, and type III collagen (*Col3)* transcript levels in the whole heart did not increase (Figure S1f). Overall, these data confirm the cardiac aging phenotype characterized by diastolic dysfunction, mild cardiac hypertrophy, early stages of inflammation, and fibrosis.

### 2.2 Autophagy is reduced in the aged heart

It has been previously reported that autophagy is decreased with age in various tissues. Therefore, we investigated whether autophagy was reduced in the aged heart by assessing levels of LC3 and the adaptor protein p62 by Western blot analysis. We used Gapdh as a loading control in the Western blot experiments after confirming that Gapdh protein levels were similar in young and aged heart tissues (Figure S2). LC3II is associated with the autophagosome membrane, and p62 is an adaptor protein that is degraded with autophagic cargo (Kabeya et al., 2000; Pankiv et al., 2007). We found that LC3II levels were significantly decreased in aged mouse hearts (Figure 1a + Figure S3), while p62 levels were significantly increased (Figure 1b + Figure S3). Moreover, immunohistochemical detection of LC3 in heart sections from young and aged mice confirmed the presence of more LC3‐positive puncta in young myocytes compared to aged myocytes (Figure 1c). Autophagy is a cellular degradation pathway involved in delivering ubiquitinated cargo to the lysosome for degradation. Therefore, we tested whether a decrease in autophagy coincided with increased levels of ubiquitinated proteins in aged hearts. Because different anti‐ubiquitin antibodies do not have equal affinities for all ubiquitin linkage types (Emmerich & Cohen, 2015), we used two different ubiquitin antibodies (polyclonal and monoclonal) in these experiments. We found that the levels of ubiquitinated proteins were elevated in the aged heart using either antibody (Figure 1d,e + Figure S3). Interestingly, the polyclonal antibody detected mostly ubiquitinated proteins with heavier mass in whole heart lysates, while the monoclonal anti‐ubiquitin preferentially detected ubiquitinated proteins with lower molecular weights. We were also intrigued by the strong broadband detected above the 50 kDa marker in the ubiquitin blot in aged mice using the monoclonal antibody (Figure 1e) because it suggests that many of the ubiquitinated proteins in aged hearts are ~50–65 kDa in size. We performed mass spectrometry analysis of the bands and identified the most abundant proteins as ATP synthase (alpha and beta) and Hsp60, as well as pyruvate kinase and tubulins (Figure S4 and Tables S1–S6). The top 100 proteins with a minimum of 40% peptide coverage identified in young (*n* = 3) and aged hearts (*n* = 3) are listed in Tables S1–S6. The proteasome is also responsible for protein degradation in cells and is another important cellular quality control pathway. Although this pathway can compensate for a decrease in autophagy, we found that proteasomal activities were similar in young and aged hearts (Figure S5). This suggests that the reduced elimination of ubiquitinated cargo is primarily due to decreased autophagic activity. The E3 ubiquitin ligase Parkin is a regulator of mitophagy, and we found that Parkin protein levels were significantly increased in the aged hearts (Figure 1f + Figure S3). Given that transcript levels of Parkin were unaltered (Figure 1f), this suggests reduced degradation of Parkin.

Next, we investigated whether elevated levels of Parkin alone could contribute to the aging process. Upon evaluation of wild‐type and cardiac‐specific Parkin transgenic (TG) mice, we found elevated levels of perivascular, but not interstitial, fibrosis, in hearts overexpressing Parkin at 16 months of age (Figure S6a). However, we found no differences in Myh7, IL‐6, and Tnf‐α transcript levels between WT and Parkin TG hearts at this age (Figure S6b). Interestingly, Parkin TG mice had increased autophagic flux and lacked accumulation of ubiquitinated proteins at this age (Figure S6c–e). This suggests that chronically elevated levels of Parkin lead to a corresponding increase in autophagic activity to balance the labeling and degradation.

Finally, we investigated whether autophagic activity was also altered with age in liver and brain tissues. Interestingly, we found that Parkin levels and protein ubiquitination were increased in both liver and brain tissues from aged mice (Figures S7 and S8). However, p62 levels were only increased in liver tissue and remained unchanged in brain tissue. There were no differences in LC3I or LC3II levels between young and aged mice in either tissue. This suggests that the reduced autophagic activity is mostly restricted to the heart at 24 months of age in these mice.

### 2.3 Autophagosome formation is reduced in the aged myocardium

**FIGURE 2:**
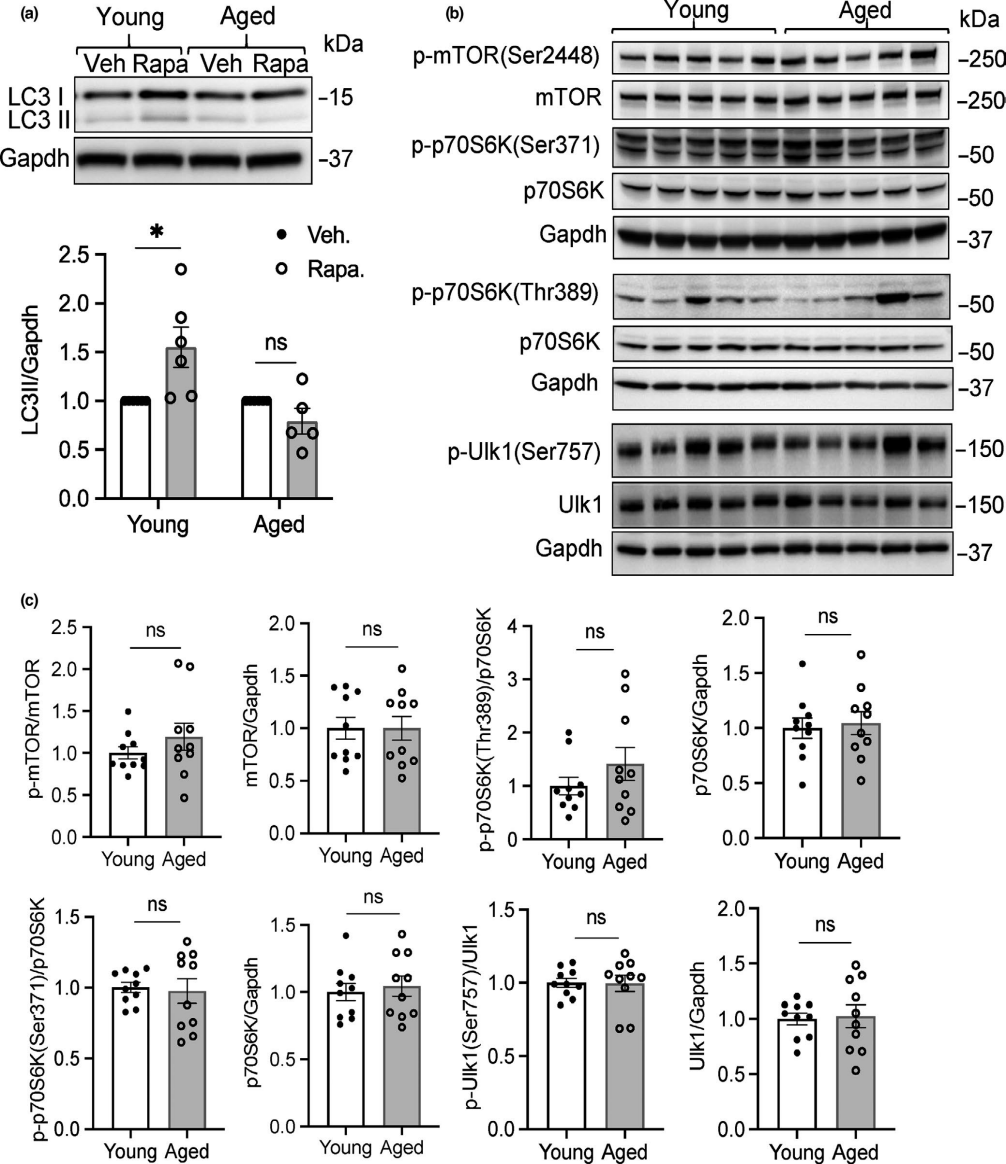
Reduced formation of autophagosomes in aged mice. (a) Representative Western blot and quantitation of LC3II levels in young and aged hearts following administration of vehicle or rapamycin (*n* = 5–6). (b) Assessment of mTOR activity in young and aged hearts. Representative Western blots of p‐mTOR(Ser2448), mTOR, p‐pS6K(Ser371 and Thr389), p70S6K, p‐Ulk1(Ser757), and Ulk1. (c) Quantitation of protein levels (*n* = 10). Data are mean ± SEM (*p < 0.05, ns = not significant)

The decreased LC3II levels indicated a reduction in autophagosome formation in the aged heart, which would contribute to reduced autophagic activity. To assess whether formation of autophagosomes was altered in the aged hearts, we monitored levels of LC3II after injecting mice with rapamycin, an inhibitor of the mammalian target of rapamycin (mTOR). mTOR functions as a negative regulator of autophagy and activated mTOR suppresses formation of autophagosomes. We found that rapamycin treatment led to an increase in LC3II in hearts of young mice, indicating increased formation of autophagosomes (Figure 2a). In contrast, the rapamycin treatment failed to increase LC3II levels in the hearts of 24‐month‐old mice. To determine whether the reduced formation of autophagosomes in the aged hearts was due to increased activation of mTOR, we examined the phosphorylation status of mTOR at Ser2448 and its downstream targets, p70S6 kinase and Ulk1 as indicators of mTOR kinase activity and signaling. We found no differences in phosphorylated mTOR (phospho‐S2448) and its substrates p70S6K (phospho‐S371 and phospho‐Thr389) or in Ulk1 (phospho‐Ser757) in young and aged hearts (Figure 2b,c). Overall, these results suggest that increased activation of mTOR is not responsible for the reduced autophagosome formation in the aged hearts.

**FIGURE 3:**
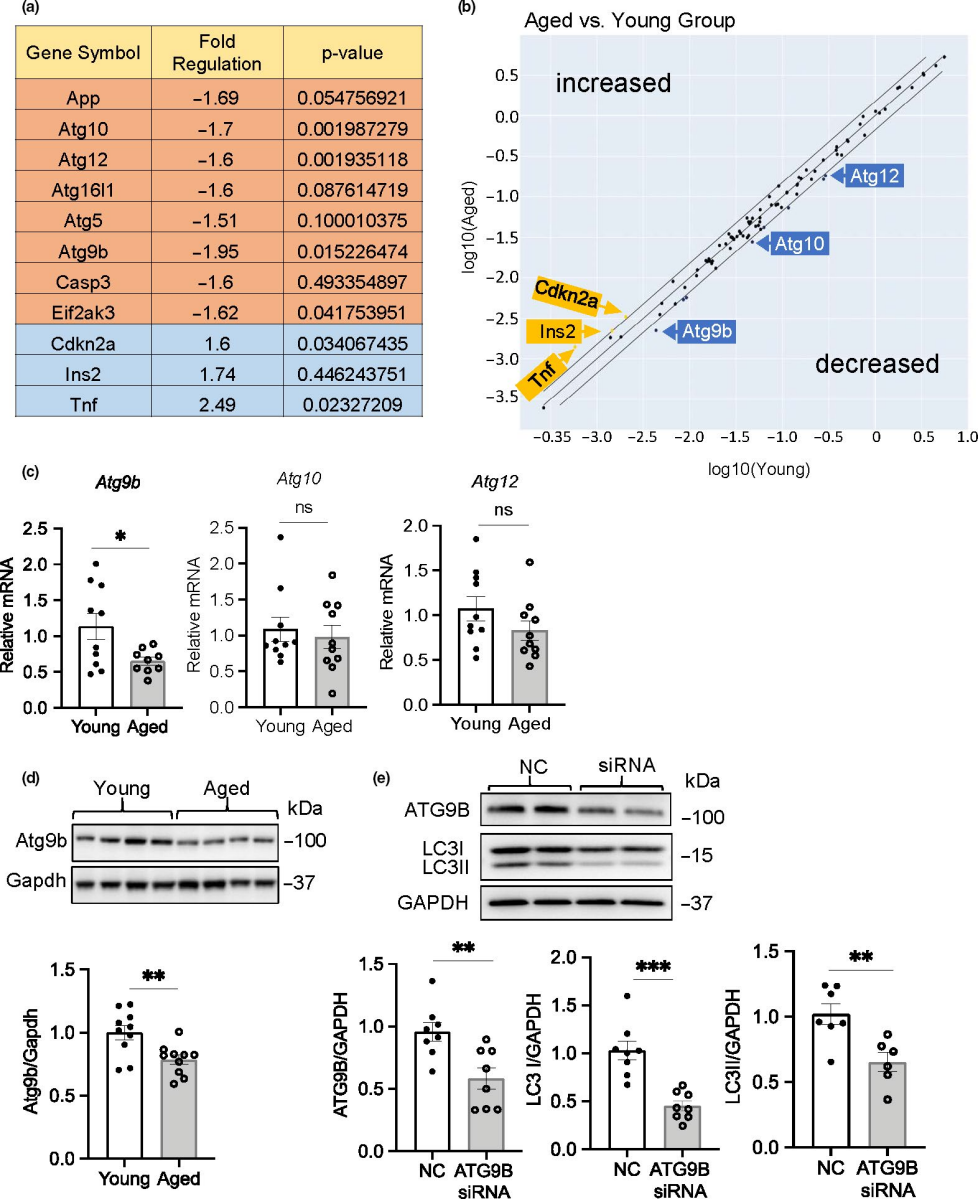
Atg9b transcript and protein levels are reduced in aged hearts. (a) Analysis of autophagy gene expression in young and aged hearts using the RT^2^ Profiler PCR autophagy gene array (*n* = 5). (b) Scatter plot for RT^2^ array analysis of genes with differential expression in aged compared to young mice. (c) Analysis of Atg9b, Atg10, and Atg12 mRNA levels by qPCR in young and aged hearts (*n* = 10). (d) Representative Western blot and quantitation of Atg9b protein levels in young and aged hearts (*n* = 10). (e) Representative Western blot and quantitation of ATG9B and LC3 levels following knockdown of *ATG9B* using siRNA in HeLa cells (*n* = 8). Data are shown as mean ± SEM (*p < 0.05, **p < 0.01, ***p < 0.001, ns = not significant)

To further explore the underlying mechanisms of the reduced autophagosome formation in aged hearts, we performed a gene expression profile analysis of key genes involved in autophagy. The mRNA profiles from young and aged hearts were screened using an autophagy PCR array containing key autophagy genes. Out of the 84 key genes involved in autophagy, we found that 3 Atg proteins involved in autophagosome formation were reduced in the aged hearts (Figure 3a,b). We confirmed that Atg9b was significantly decreased in the aged hearts both at the mRNA and protein levels compared to young hearts (Figure 3c,d). We were unable to confirm reduced transcript levels of Atg10 and Atg12 by traditional qPCR (Figure 3c). Atg9b is involved in delivering membrane to the expanding phagophore (Yamamoto et al., 2012). It has previously been reported that Atg9 is critical for autophagy (Yamamoto et al., 2012) and knockdown of *ATG9B* in HeLa cells using siRNA confirmed that reduced ATG9B protein leads to reduced autophagy (Figure 3e). Overall, these data suggest that the compromised autophagy in aged hearts is due to decreased levels of proteins involved in autophagosome formation/expansion.

### 2.4 Reduced autophagy coincides with an increase in mitochondria that have been labeled for mitophagy

**FIGURE 4:**
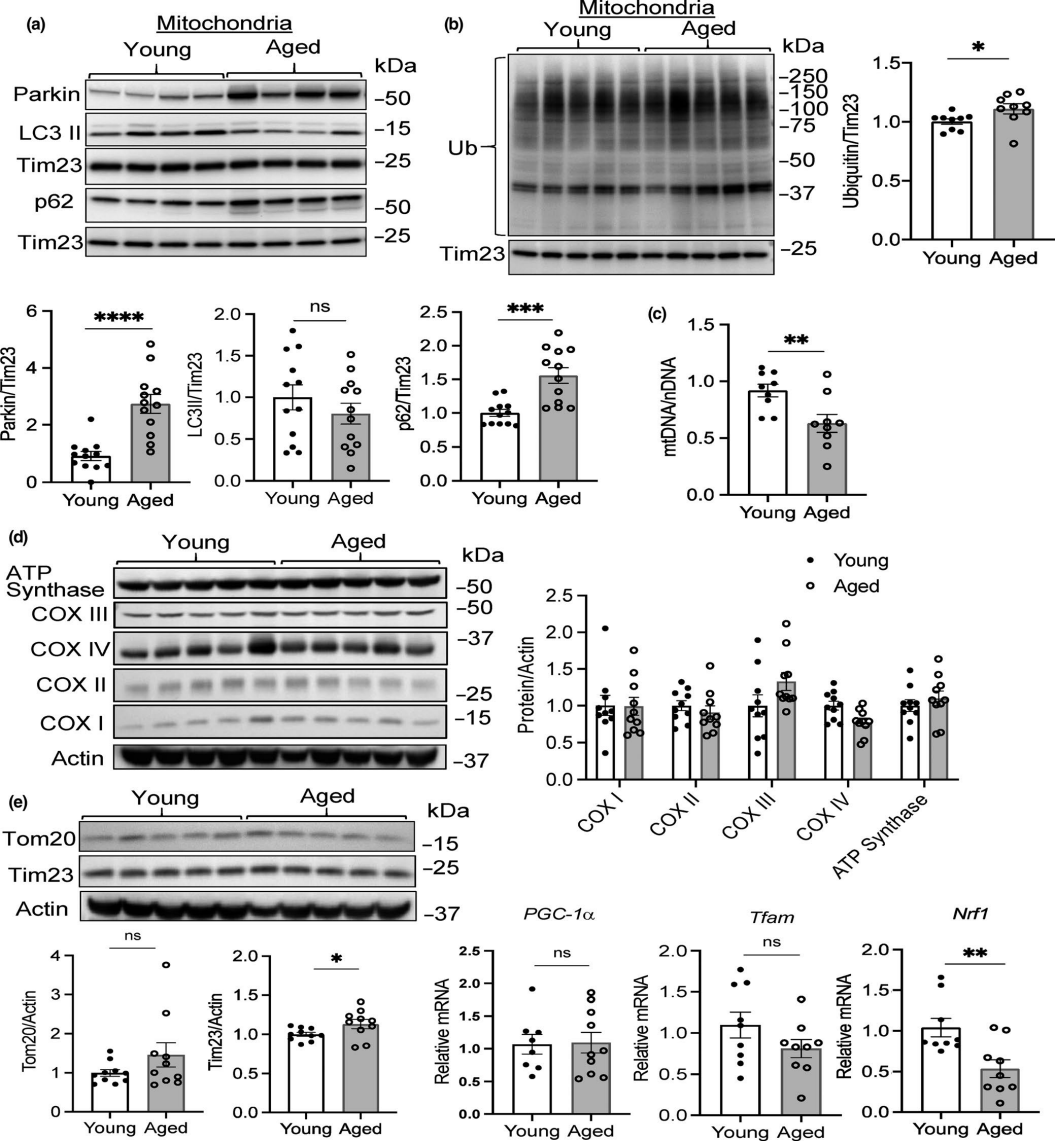
Reduced mitochondrial turnover in aged hearts. Representative Western blots and quantitation of (a) Parkin, LC3, p62, and (b) ubiquitin levels in the mitochondrial fraction from young and aged hearts (*n* = 9). Ubiquitin was detected using a polyclonal antibody. (c) Mitochondrial DNA (mtDNA) content in young and aged hearts (*n* = 11). (d) Representative Western blots and quantitation of proteins involved in mitochondrial oxidative phosphorylation: COX I = complex I subunit NDUFB8; COX II = complex II subunit 30 kDa; COX III = complex III subunit core 2; COX IV = complex IV subunit II; ATP synthase = ATP synthase subunit α (*n* = 9–10). (e) Representative Western blots and quantitation of mitochondrial proteins Tim23 and Tom20 (*n* = 10). (f) Analysis of PGC‐1α, Tfam, and Nrf1 mRNA levels by qPCR in young and aged hearts (*n* = 8–10). Data are shown as mean ± SEM (*p < 0.05, **p < 0.01, ***p < 0.001, ****p < 0.0001 ns = not significant)

Given that aged mice had a decrease in general autophagy and increased Parkin levels in aged hearts, we investigated whether the aged mitochondria had been labeled for mitophagy. We found significant increases in Parkin and p62 levels in the mitochondrial fraction from aged hearts (Figure 4a). Western blotting using a polyclonal ubiquitin antibody showed a small but significant increase in ubiquitinated mitochondrial proteins (Figure 4b). Ubiquitination of proteins in the mitochondrial fraction trended higher but did not reach significance when using a monoclonal anti‐ubiquitin (Figure S9). The elevated Parkin, p62, and ubiquitin levels indicate that there is an increase in mitochondria that have been labeled for degradation. Consistent with reduced formation of autophagosomes, LC3II levels did not increase in the aged mitochondrial fraction (Figure 4a). These findings suggest that the rate of mitochondrial turnover is reduced due to decreased autophagosome formation.

Therefore, we investigated whether the reduced autophagy had an effect on mitochondrial content in the aged hearts. Changes in mitochondrial DNA (mtDNA) content, measured as mitochondrial genome‐to‐nuclear genome ratio (mtDNA/nDNA) using real‐time quantitative PCR, often correlates with changes in mitochondrial mass. Interestingly, we found that mtDNA content was significantly reduced in the aged hearts (Figure 4c), suggesting a potential reduction in mitochondrial number. However, Western blotting for various mitochondrial proteins involved in oxidative phosphorylation (ATP synthase, subunits in complexes IV, III, II, and I) and protein import (Tom20, Tim23), showed similar levels of these proteins in young and aged hearts (Figure 4d,e). This suggests that there is no change in mitochondrial content but that the mitochondrial genome is unstable in the aged hearts. To ensure a stable mitochondrial mass, mitophagy is balanced by mitochondrial biogenesis to replace degraded mitochondria. Pgc‐1α, Tfam, and Nrf1 are regulators of mitochondrial biogenesis (Li, Hou, & Hao, 2017), and we found that Pgc‐1α and Tfam transcript levels were similar in young and aged hearts (Figure 4f). In contrast, mRNA levels of Nrf1, a major transcriptional regulator of mitochondrial biogenesis, were significantly reduced in the aged hearts. A simultaneous decrease in mitophagy and mitochondrial biogenesis prevents a change in overall mitochondrial content.

**FIGURE 5:**
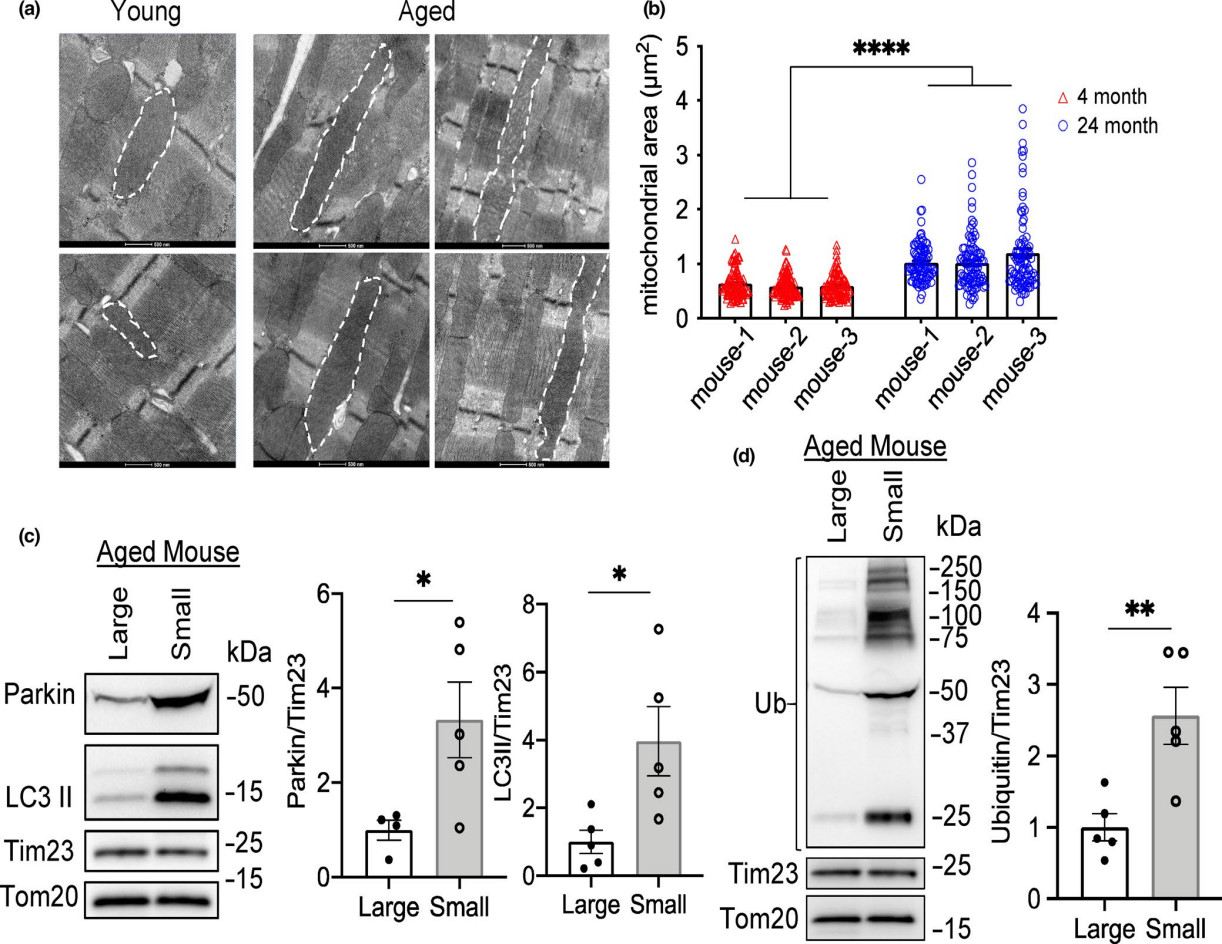
Megamitochondria are present in aged hearts. (a) Representative transmission electron micrographs of young and aged heart tissues reveal the presence of enlarged mitochondria in the hearts of aged mice. Scale bars are 500 nm. (b) Analysis of mean mitochondrial area (μm^2^) in young and aged myocytes. One hundred mitochondria per heart were scored (*n* = 3). Representative Western blots and quantitation of (c) Parkin, LC3II, and (d) ubiquitin levels in large and small mitochondria from aged hearts separated by differential centrifugation (*n* = 5). A monoclonal antibody was used to detect ubiquitin levels. (*p < 0.05, **p < 0.01, ****p < 0.0001).

To further assess changes in the myocardium at the ultrastructural level, we evaluated heart sections prepared from young and aged mice using transmission electron microscopy (TEM). We focused on identifying changes in aged myocytes since they are postmitotic and cannot be replaced when lost. Interestingly, we found that many of the mitochondria were enlarged in the aged myocytes and that the average volume of mitochondria was significantly increased compared to young myocytes (Figure 5a,b). Since we observed increased Parkin at the mitochondria in the aged hearts when analyzing a mix of normal and enlarged mitochondria, we wanted to determine whether there was a difference in the labeling of normal/small versus large mitochondria in the aged hearts. Using differential centrifugation, we separated the enlarged and small/normal mitochondria from the aged hearts and then analyzed the protein content in two different mitochondrial fractions by Western blotting. Analysis of mitophagy markers showed that the small mitochondria contained increased levels of Parkin, LC3II, and ubiquitination when compared to enlarged mitochondria (Figure 5c,d), suggesting that smaller fragmented mitochondria are selectively targeted for degradation. Levels of other mitochondrial proteins, such as Tom20 and Tim23, were found to be similar in enlarged and small mitochondria (Figure 5c,d).

### 2.5 Mitochondrial fission is reduced in the aged hearts

**FIGURE 6:**
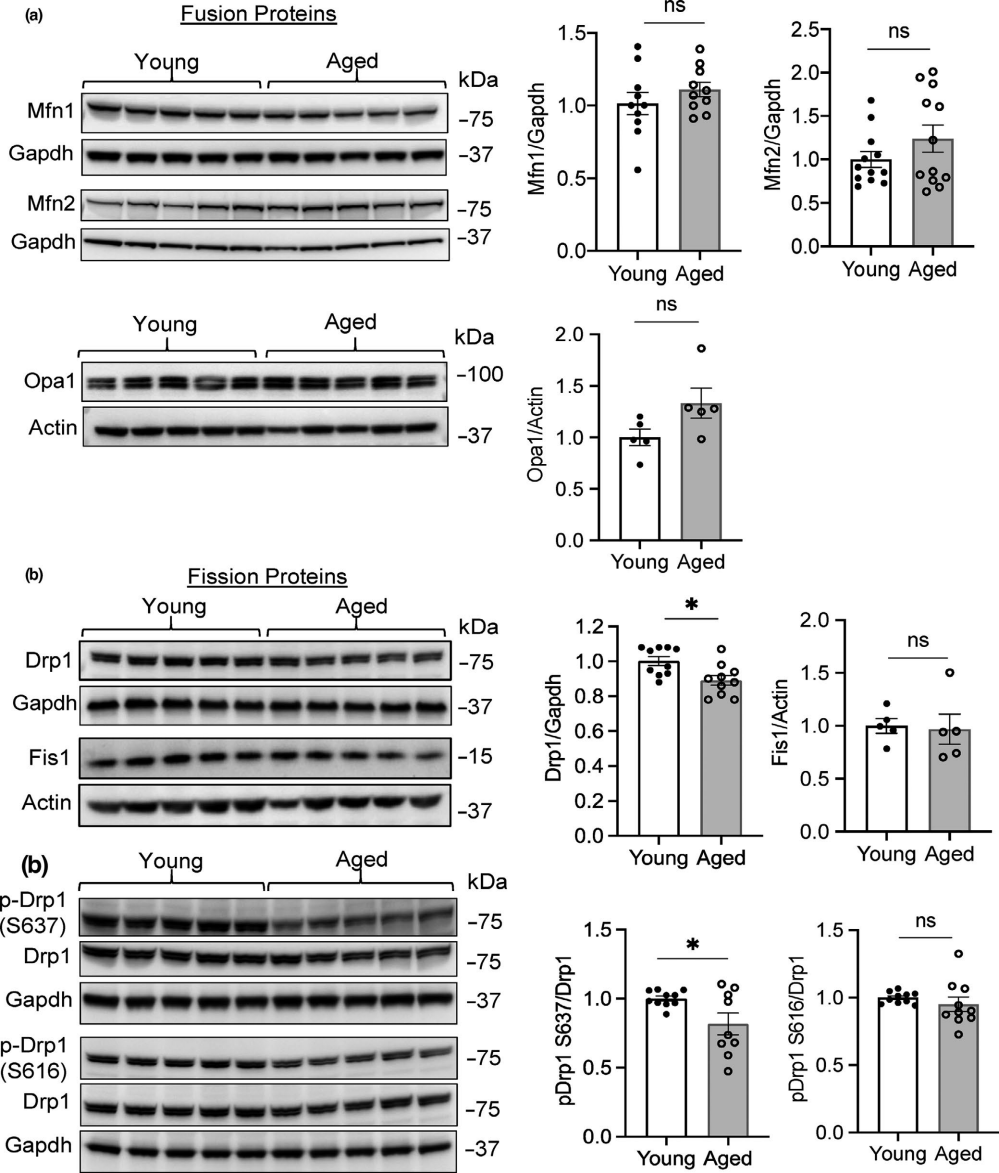
Mitochondrial fission is reduced in aged hearts. (a) Representative Western blots and quantitation of mitochondrial fusion proteins Mfn1, Mfn2, and Opa1 in whole heart lysates from young and aged mice (*n* = 5–10). (b) Representative Western blots and quantitation of mitochondrial fission proteins Drp1 and Fis1 in whole heart lysates from young and aged mice (*n* = 5–10). (c) Representative Western blots and quantitation of the phosphorylation status of Drp1 at Ser637 and Ser616 in young and aged hearts (*n* = 9–10). Data are mean ± SEM (*p < 0.05, ns = not significant)

A change in mitochondria morphology is a highly regulated process, and the balance between fission and fusion dictates the overall morphology of the mitochondria. Based on the presence of enlarged mitochondria in the aged hearts, we examined whether there were differences in key regulators of mitochondrial fission and fusion. Mitofusin 1 and 2 (Mfn1/2) are involved in fusion of the outer mitochondrial membrane, while Opa1 regulates fusion of the inner membranes. However, we found similar levels of Mfn1, Mfn2, and Opa1 in young and aged hearts (Figure 6a). Drp1 and Fis1 regulate mitochondrial fission, and we found a significant decrease in Drp1 in aged hearts (Figure 6b). Fis1 levels were unchanged. Drp1 activity is regulated by phosphorylation on at least two distinct residues. Phosphorylation at Ser637 inhibits Drp1 activity, while phosphorylation at Ser616 activates Drp1. Interestingly, we found that the phosphorylation status of Drp1 was also altered in the aged hearts. While Drp1 was dephosphorylated at Ser‐637 in aged hearts, there was no increase in the phosphorylation of Ser616 (Figure 6c). Overall, these results suggest that a decrease in Drp1 levels combined with a potential imbalance in the phosphorylation status of Drp1 prevents its activation and shifts the mitochondrial morphology toward a fused state.

## 3 DISCUSSION

The molecular mechanisms underlying the defective autophagy with age remain poorly characterized, especially in the heart. The findings in this study provide important new insights into the mechanism underlying the insufficient autophagy in aged hearts and the potential downstream consequences on mitochondrial turnover and morphology. Our findings suggest that the suppressed autophagic activity is, at least in part, due to decreased levels of key autophagy‐related proteins such as Atg9b, which leads to reduced ability to form autophagosomes. We also discovered a concurrent accumulation of the E3 ubiquitin ligase Parkin and ubiquitinated proteins in the aged heart. Finally, our data demonstrate reduced Drp‐1‐mediated fission and formation of enlarged mitochondria in the aged heart. Overall, our findings suggest that there is an imbalance in the labeling and degradation steps in the aged myocardium due to reduced formation of autophagosomes.

In our screen for changes in autophagy regulators, we discovered that Atg9b transcript and protein levels were significantly reduced in the aged heart. Atg9 is a transmembrane protein and is required for autophagy. Atg9‐positive vesicles supply the growing autophagosome with membrane and other required components (Imai et al., 2016; Yamamoto et al., 2012). There are two different isoforms of Atg9 in vertebrates: Atg9a and Atg9b. The differences between these two distinct isoforms are currently unclear, but there is evidence that they have different tissue‐specific functions. For instance, Atg9b has been linked to various pathologies, including hepatocellular carcinoma (HCC), and a combination of suppressed Atg9b expression and decreased autophagy correlates with poor prognosis for patients with HCC (Wang, Tan, Li, & Feng, 2017). A genetic screen in flies for autophagy proteins involved in regulating health and life spans revealed that RNAi knockdown of Atg9 led to reduced survival (Xu et al., 2019). Specific knockdown of Atg9 in fly hearts led to accelerated age‐dependent loss of cardiac function and development of hypertrophy (Xu et al., 2019), confirming the importance of Atg9 in the heart and preventing the aging process in flies.

Previous studies have demonstrated that reduced autophagy leads to accumulation of cargo, which includes protein aggregates and dysfunctional mitochondria (Pattison, Osinska, & Robbins, 2011; Pyo et al., 2013). Consistent with this, we observed an increase in the levels of ubiquitinated proteins in the aged hearts, which is likely due to their reduced clearance via autophagy as proteasomal activity was unaltered. Interestingly, we also found increased levels of Parkin protein, but not transcripts, in aged hearts indicating that the increased Parkin protein in the aged hearts might be due to reduced degradation. Our data also suggest that elevated levels of Parkin might not be beneficial unless there is a corresponding increase in autophagy to clear ubiquitinated substrates. Despite enhanced autophagic activity in Parkin TG hearts, there was still increased perivascular fibrosis in these hearts at 16 months of age. Hoshino et al. previously reported that transgenic mice with overexpression of Parkin in the heart lead to preserved mitochondrial function in the aged heart (Hoshino et al., 2013). The underlying reasons for these different findings are likely due to the different genetic backgrounds of the transgenic mice (C57BL/6 vs. C57BL/6J) and the level of Parkin overexpression. The mice used in our study have much higher levels of Parkin, thus creating a greater imbalance between labeling of cargo and degradation. This could potentially overwhelm both the UPS and the autophagy degradation pathways in some cells in the aging heart. Importantly, our findings demonstrate that without a corresponding increase in autophagosome formation, elevating Parkin levels alone will not be beneficial and could potentially accelerate the aging process when the imbalance (labeling vs. degradation) reaches a certain threshold.

A decline in mitochondrial function in myocytes is also thought to be a contributor to cardiac aging (Dai & Rabinovitch, 2009). Here, we found that reduced autophagy coincided with formation of elongated mitochondria in cells. The presence of enlarged mitochondria has previously been noted in various tissues with age, including cardiac myocytes (Coleman, Silbermann, Gershon, & Reznick, 1987; Tandler, Dunlap, Hoppel, & Hassan, 2002), skeletal muscle (Beregi & Regius, 1987), and neurons (Vanneste & van den Bosch de Aguilar, 1981). It remains unknown whether these enlarged mitochondria are beneficial for the heart or whether they contribute to the development of cardiac aging and disease. It is possible that the enlarged mitochondria are formed as an adaptive response to the reduced autophagy in the aged hearts, but this still needs to be investigated in more detail. A recent study reported that flies with heart‐specific Atg9 knockdown had reduced autophagy, which led to formation of elongated mitochondria with age in the heart (Xu et al., 2019), confirming a link between reduced autophagy and increased mitochondrial fusion. Fusion between damaged and healthy mitochondria leads to mixing of damaged components and dilution of the damage (Chen et al., 2010). Under normal conditions, damaged mitochondria undergo asymmetrical fission where the dysfunctional mitochondrial fragments are then eliminated by mitophagy. However, if these mitochondria fail to be eliminated by autophagosomes, then they can fuse with healthy mitochondria instead (Song, Mihara, Chen, Scorrano, & Dorn, 2015). Although the increased mitochondrial fusion might function to dilute mitochondrial damage, eventually the damage will lead to excessive contamination of the mitochondrial pool. As aging progresses, an increasing proportion of mitochondria will reach a threshold for damage where they will no longer function properly. Thus, it will be important for future studies to dissect the relationship between reduced autophagy and formation of enlarged mitochondria in the aging heart.

A limitation of the current study is that we only assessed autophagy markers and mitochondrial proteins in the intact heart. The heart is composed of many cell types, including cardiac myocytes, fibroblasts, endothelial cells, and perivascular cells. The immunostaining of heart sections confirmed a reduced number of for LC3‐positive autophagosomes in aged cardiac myocytes. Similarly, TEM analysis confirmed changes in mitochondrial morphology in aged myocytes. Whether autophagic activity and/or mitochondrial function are altered in non‐myocyte cells still needs to be investigated. Myocytes occupy ~70%–85% of the volume of the mammalian heart (Zhou & Pu, 2016), but it is clear that the non‐myocytes are also important contributors to myocyte contraction and cardiac homeostasis. Thus, it is likely that changes in autophagy and mitochondrial function in both myocytes and non‐myocytes contribute to the cardiac aging process. Future studies need to focus on examining the effect of aging on autophagy and mitochondria in the various cell types in the heart.

In summary, our study has uncovered that the formation of autophagosomes is reduced in aged hearts, which results in an imbalance between labeling and degradation of cargo such as mitochondria. The reduction in autophagosome formation is, in part, due to decreased levels of the autophagy‐related protein Atg9b. Thus, our findings highlight the potential feasibility of targeting specific autophagy regulators (i.e., Atg9b levels), directly or indirectly, to correct the defect and consequently restore normal autophagic activity in aging tissues. Future studies need to focus on why Atg9b is reduced with age and whether this can be prevented or restored.

## 4 EXPERIMENTAL PROCEDURES

### 4.1 Animals

All animal experiments were performed in accordance with the National Institutes of Health Guidelines on the Use of Laboratory Animals and were approved by the Institutional Animal Care and Use Committee of the University of California, San Diego. Four‐month‐old (young) and 24‐month‐old (aged) male C57BL/6 mice (a total of 60 in each group) were obtained from the National Institute of Health (NIH) Institute of Aging colony (Charles River). The cardiomyocyte‐specific Parkin transgenic (TG) mice were generated on a C57BL/6 background and have been previously described (Woodall et al., 2019). Mice were housed under standard conditions and given free access to chow and water with a 12/12‐hr dark–light cycle. Echocardiography was performed on young and aged mice using a Vevo770 In Vivo Micro‐Imaging System with an RMV707B 15–45 MHz imaging transducer (VisualSonics Inc.) as previously described (Thomas et al., 2013). M‐mode, B‐mode, and pulsed wave Doppler views were acquired. Mice were kept on a recirculating water warming pad and maintained under light anesthesia (0.5%–1% isoflurane, 98%–99.5% O_2_), while measurements were obtained. The VisualSonics software was used for analysis and quantification. Tissues were collected from mice following anesthesia with pentobarbital (100 mg/kg) and exsanguination. For the in vivo rapamycin autophagy experiments, 3 mg/kg rapamycin (LC Laboratories) was administered to young and aged mice by intraperitoneal injection, and then, a second dose was administered 12 hr later. The control mice were injected with 10% DMSO in saline as the vehicle. Hearts were harvested 24 hr following the initial injection. After harvest, the atria were removed and the ventricles were rinsed in ice‐cold sterile PBS to remove remaining blood. The ventricles were snap‐frozen at −80°C and saved for qPCR analysis and Western blotting.

### 4.2 Histology and transmission electron microscopy

Hearts were arrested in diastole with 200 mM KCl, fixed in 10% neutral buffered formalin for 24 hr, and then dehydrated in 70% alcohol for 24 hr before being processed in a tissue processor (Thermo Scientific STP 120). Hearts were embedded in paraffin and cut into 6‐μm sections using a microtome (Leica Biosystems). Deparaffinized and rehydrated sections were stained with haematoxylin and eosin (H&E) or Masson's trichrome (MilliporeSigma). Alternatively, deparaffinized and rehydrated heart sections were subjected to antigen retrieval in sodium citrate buffer (10 mM sodium citrate, 0.05% Tween‐20, pH 6.6), blocked with normal goat serum, incubated with anti‐LC3 (Cell Signaling, #4108) overnight at 4°C, followed by incubation with Alexa Fluor 488 secondary antibodies (Life Technologies) for 90 min at room temperature. The sections were mounted with VECTASHIELD HardSet Mounting Media with DAPI (Vector Laboratories). For each experiment, all images were collected with the same exposure time using a Nikon Eclipse microscope equipped with DS‐Fi3 camera (for color images) or a DS‐Qi2 (for fluorescence images). The Masson's trichrome blue stain and total tissue area were quantified in ImageJ. Percent positive signal was determined for each image (*n* = 4–6 images per heart using the 20× objective), and mean % positive signal was calculated per heart.

Transmission electron microscopy was performed on heart sections from 3 young and 3 aged mice as previously described (Orogo et al., 2015). Hearts were subjected to fixation with 2.5% glutaraldehyde in 0.1 M cacodylate buffer, followed by post‐fixation in 1% osmium tetroxide. Hearts were then treated with 0.5% tannic acid and 1% sodium sulfate and cleared in 2‐hydroxypropyl methacrylate. Finally, hearts were embedded in LX112 (Ladd Research). A Philips CM100 electron microscope (FEI) was used to examine sections after they were mounted on copper slot grids coated with Parlodion and stained with uranyl acetate and lead citrate. Mean mitochondrial area (μm^2^) was quantified from measurements of 100 mitochondria per heart.

### 4.3 Real‐time quantitative PCR

For gene expression assays, the RNeasy Fibrous Tissue Mini Kit (Qiagen) was used to extract RNA from tissue. The QuantiTect Reverse Transcription Kit (Qiagen) or RT^2^ First‐Strand Kit (Qiagen) was used for the synthesis of cDNA. TaqMan primers were obtained from Life Technologies/Thermo Fisher Scientific for *Park2*, *Myh7*,* Pgc*‐*1α*,* IL*‐*6*,* Tnfα*,* Atg9b*,* Atg10*,* Atg12*,* Tfam*,* Nrf1*,* Tgfβ*,* Collagen I*,* Collagen III*, and *Rn18s*. The TaqMan Universal Master Mix II was purchased from Applied Biosystems/Life Technologies. Autophagy RT2 Profile PCR Arrays were obtained from Qiagen. A CFX96 Real‐Time PCR Detection System (Bio‐Rad) was used to perform qPCR. To calculate fold change in gene expression, relative amounts of mRNA were normalized to *Rn18s* and the 2^(−ΔΔCt)^ method was employed. To assess mitochondrial DNA copy number, genomic DNA was extracted from young and aged hearts using the GenElute Mammalian Genomic DNA Miniprep Kit (Sigma) and PCR‐amplified with TaqMan Universal Master Mix II. 18S rRNA was used as a control for nuclear DNA content, and D‐loop was used for mtDNA quantitation as previously described (Woodall et al., 2019).

### 4.4 Western blot

Ventricles were minced and then homogenized in lysis buffer composed of 50 mM Tris‐HCl, 150 mM NaCl, 1 mM EGTA, 1 mM EDTA, complete protease inhibitor cocktail (Roche), PhosSTOP (Roche), and n‐ethylmaleimide (Sigma). After the addition of Triton X‐100 (1% final concentration), the homogenates were incubated on ice for 45 min and then cleared by centrifugation at 20,000 *g* for 20 min. To isolate mitochondrial fractions, ventricles were minced in buffer containing 250 mM sucrose, 5 mM KH_2_PO_4_, 2 mM MgCl_2_, 10 mM MOPS, pH 7.4, 1 mM EGTA, 0.1% fatty acid‐free BSA, protease inhibitor cocktail (Roche), PhosSTOP (Roche), and n‐ethylmaleimide (Sigma). The tissue was briefly homogenized by polytron followed by 3–4 strokes using the Potter–Elvehjem Teflon tissue grinder. Unbroken cells, debris, and nuclei were removed by centrifugation at 600 *g* for 3 × 5 min at 4°C. To obtain the mitochondrial fraction, the supernatant was centrifuged at 6,000 *g* for 10 min at 4°C. For separation of large vs. small mitochondria in aged hearts, large mitochondria were collected by centrifugation at 2,000 *g*, while the remaining small/normal mitochondria were spun down at 6,000 *g*. The final mitochondrial pellets were resuspended in 50 mM Tris‐HCl, 150 mM NaCl, 1 mM EGTA, 1 mM EDTA, 1% Triton X‐100, protease inhibitor cocktail (Roche), PhosSTOP (Roche), and n‐ethylmaleimide (Sigma). All protein concentrations were determined by Bradford assay.

The following antibodies were purchased to probe the membranes: Actin (GeneTex, GTX109638), Atg9b (Abcam, ab117591 or Novus NBP1‐77169), Drp1 (BD Biosciences, #611113), Fis1, (Abcam, ab96764), GAPDH (GeneTex, GTX627408), LC3 (Cell Signaling, #4108), Mfn1 (Santa Cruz Biotechnology, sc‐50330), Mfn2 (MilliporeSigma, M6319), MitoProfile Total OXPHOS Rodent WB Antibody Cocktail (Abcam/MitoSciences, MS604), mTOR (Cell Signaling, #2983), Opa1 (BD Biosciences, #612607), p62 (Abcam, ab56416), p70S6 Kinase (Cell Signaling, #49D7), Parkin (Prk8) Monoclonal antibody (Cell Signaling, #4211), Parkin Polyclonal antibody (Cell Signaling, #2132S), Phospho‐Drp1 S616 (Cell Signaling, #3455), Phospho‐Drp1 S637 (Cell signaling, #6319), Phospho‐mTOR Ser2448 (Cell Signaling, #5536), Phospho‐p70 S6 Kinase Ser371 (Cell Signaling, #9208), Phospho‐p70 S6 Kinase Thr389 (Cell Signaling, #9234), Phospho‐Ulk1 Ser757 (Cell Signaling, #6888), Tim23 (BD Biosciences, #611222), Tom20 (Santa Cruz, sc‐11415), Tubulin (MilliporeSigma, T6074), Ubiquitin Monoclonal antibody (Santa Cruz Biotechnology, sc‐8017), Ubiquitin Polyclonal antibody (Cell Signaling, #3933), and Ulk1 (Cell Signaling, #8054). Blots were developed with the SuperSignal West Dura Extended Duration Substrate (Thermo Fisher, #34076) and images captured using a ChemiDoc XRS+ System (Bio‐Rad). To quantify protein bands, grayscale images of blots were either analyzed using Image‐Lab software (Bio‐Rad) or imported into ImageJ (NIH). A frame was drawn around each band in the same row to select the region of interest. The same frame was used for each protein band across one row. The pixel density for each band was normalized to the pixel density of the corresponding loading control.

### 4.5 Proteasomal activity assay

Ventricles were homogenized by polytron in assay buffer containing 50 mM HEPES, 10 mM NaCl, 1.5 mM MgCl_2_, 1 mM EDTA, 1 mM EGTA, 250 mM sucrose, 2 mM ATP, and 5 mM DTT, pH 7.8. The homogenates were centrifuged at 16,000 *g* for 10 min, and the resulting supernatant was collected and used to assay proteasomal activities. Ten μg of protein/well was added to a clear‐bottomed/black‐walled 96‐well plate. One hundred ninety‐five μl assay buffer and 5 μl of fluorescent substrate (chymotrypsin‐like, trypsin‐like, or caspase‐like, Enzo) were also added to each well. Plates were incubated at 37°C for 60 min, and then, fluorescence was measured at A_360_ex/A_460_em on a fluorescent plate reader. Each sample was measured in triplicate.

### 4.6 Cell culture and siRNA knockdown experiments

HeLa cells were cultured in media consisting of DMEM (Life Technologies) supplemented with 10% fetal bovine serum (Life Technologies), 100 U/ml penicillin (Gemini), and 100 μg/ml streptomycin (Gemini) and cultured at 37°C in a 5% CO_2_ atmosphere. ATG9B knockdown was performed by transfecting HeLa cells with 100 nM MISSION small interfering RNA (siRNA) Universal Negative Control #1 (Sigma, SIC001) or ATG9B siRNA (Sigma, SASI_Hs02_00368624) using Lipofectamine RNAiMax Transfection Reagent (Invitrogen) according to the manufacturer's instructions. After 48 hr, cells were harvested for Western blot analysis.

### 4.7 Proteomics analysis

The proteomics analysis was done in collaboration with the Biomolecular/Proteomics Mass Spectrometry Facility at UCSD as described (Shevchenko, Wilm, Vorm, & Mann, 1996). Briefly, proteins were subjected to in‐gel tryptic digestion and the peptides were analyzed by ultra‐high‐pressure liquid chromatography (UPLC) coupled with tandem mass spectroscopy (LC‐MS/MS) using nanospray ionization. The nanospray ionization experiments were performed using an Orbitrap fusion Lumos hybrid mass spectrometer (Thermo) interfaced with nanoscale reversed‐phase UPLC (Thermo Dionex UltiMate™ 3000 RSLC nano System). Protein identification and label‐free quantification were carried out using PEAKS Studio 8.5 (Bioinformatics Solutions Inc.)

### 4.8 Statistical analyses

Data are expressed as mean ± standard error of mean (SEM). Because the two groups (young and aged) in this study were all males from same genetic background and environment, we used Student's *t* test or Mann–Whitney *U* test to evaluate differences between the means (GraphPad Software, Inc., USA). Data that passed the normality test were compared using an unpaired Student's *t* test. A nonparametric Mann–Whitney *U* test was used for data that were not normally distributed. A *p*‐value < 0.05 was considered significant.

## CONFLICT OF INTEREST

Authors declare that there is no conflict of interest in this project.

## AUTHORS' CONTRIBUTION

ÅBG, WJL, and AGM designed the study, analyzed the experiments, and wrote the paper. WJL and AGM designed, performed, and analyzed the majority of the experiments. MAL performed the echocardiography on the mice. RYD and RHN assisted with Western blotting and qPCR experiments. All authors reviewed the results and approved the manuscript.

## Supporting information

Appendix S1

## Data Availability

The data that support the findings of this study are available from the corresponding author upon reasonable request.
