# Supplementary material for: Aging is associated with a decline in Atg9b‐mediated autophagosome formation and appearance of enlarged mitochondria in the heart
Source: Aging Cell. 2020 Jul 6;19(8):e13187. doi: 10.1111/acel.13187 (PMC7431832; doi:10.1111/acel.13187)
Supplement: Supplementary file 1 — Appendix S1 [file ACEL-19-e13187-s001.pdf]

Liang et al., Supplemental information
Figure S1

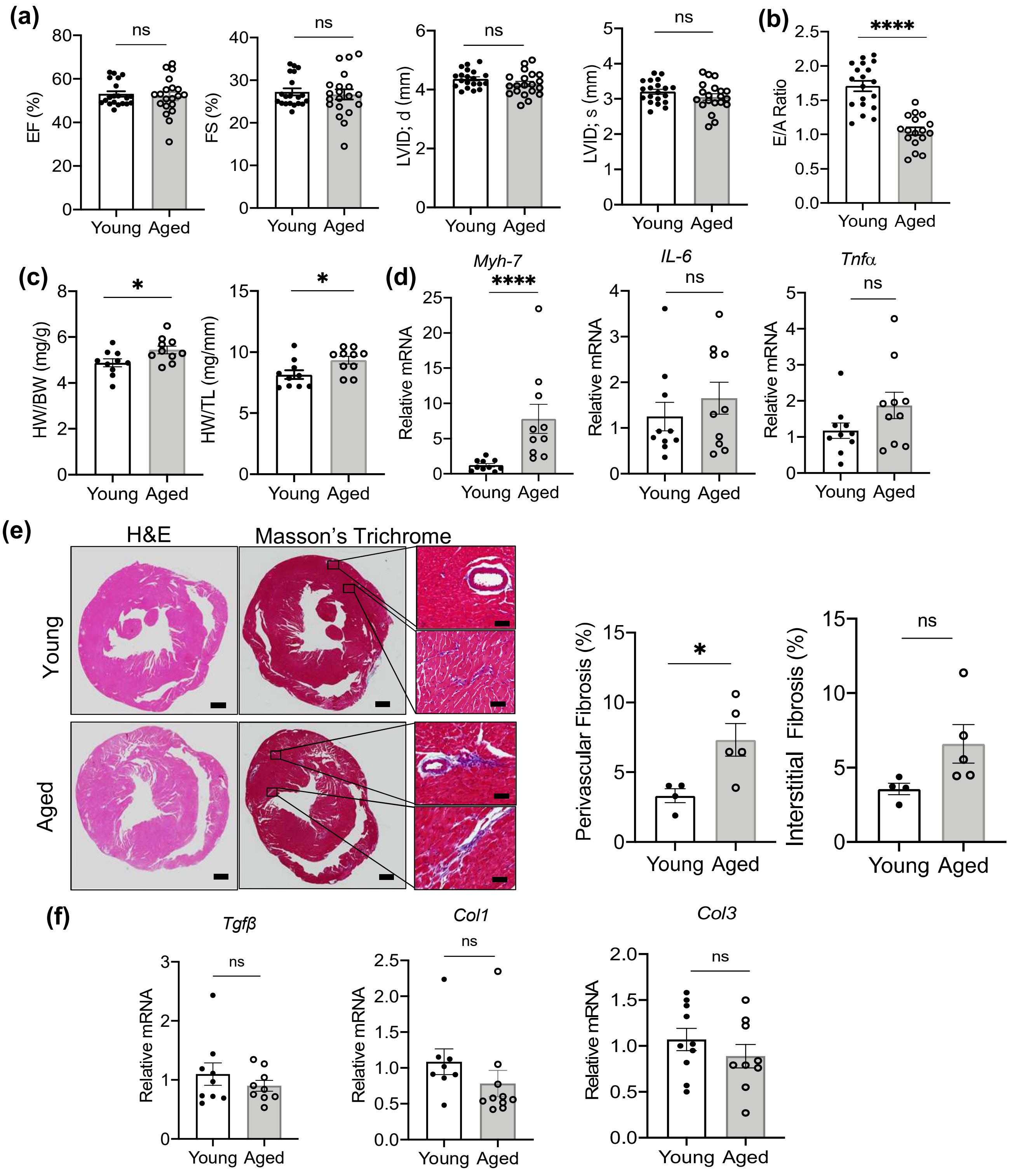

Figure S1 cont.

Figure S1. Characterization of young (4-month) and aged (24-month) male mice. (a) Echocardiography shows no significant differences in ejection fraction (EF), fractional shortening (FS), left ventricular internal dimension at diastole (LVID;d) and systole (LVID;s) (n=20). (b) E/A ratio was significantly reduced in aged mice (n=20). (c) Assessment of the ratio of heart weight compared to body weight (HW/BW) and heart weight versus tibia length (HW/TL) in young and aged mice (n=10). (d) Analysis of *Myh7, IL-6* and *TNFα* mRNA levels by qPCR in young and aged hearts (n=10). (e) Representative images of young and aged hearts stained with H&E and Masson's trichrome. Quantitation of perivascular and interstitial fibrosis in heart tissue from young and aged mice (n=4-5). (f) Analysis of fibrosis markers Transforming growth factor beta (*Tgfβ)*, Collagen 1 (*Col1)*, and Collagen 3 (*Col3)* mRNA levels by qPCR in young and aged hearts (n=10). Scale bars are 500 μm and 50 μm. Data represent mean $\pm$ SEM (*P<0.05,****P<0.0001, ns = not significant).

Figure S2

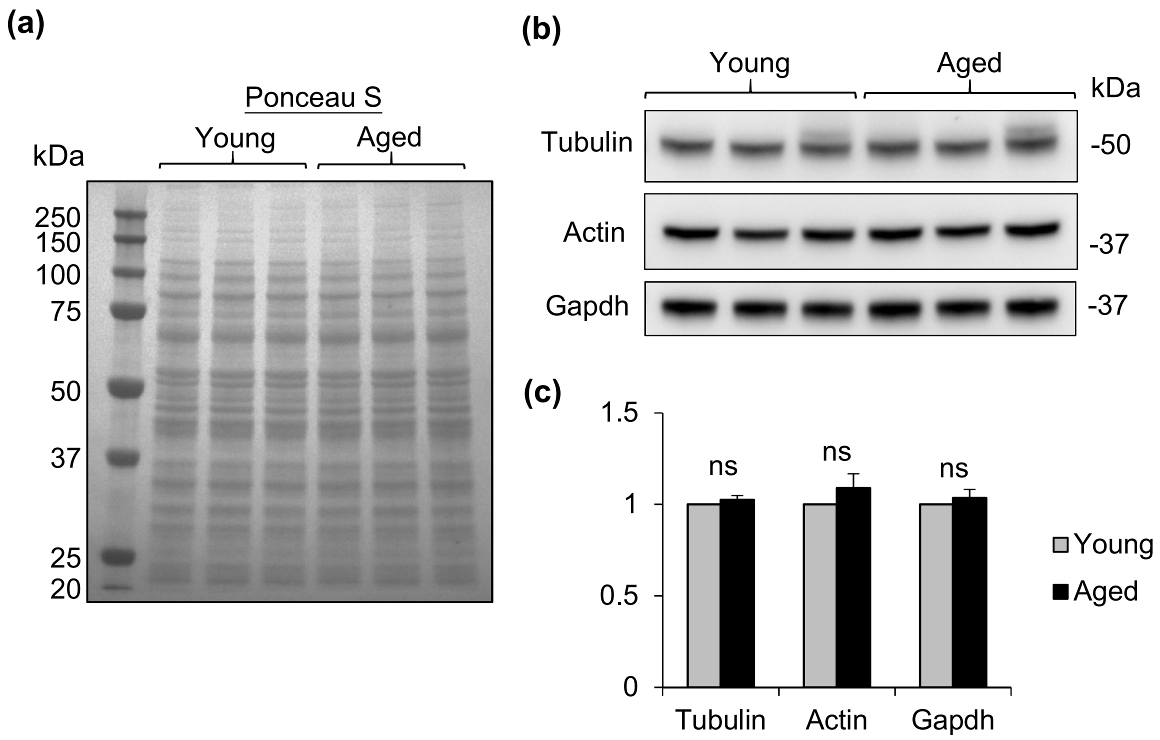

Figure S2. Protein levels of three different standard loading controls in young (4 month) and aged (24 month) hearts. **(a)** Ponceau S stain of membrane to confirm equal loading of proteins. **(b)** Western blot analysis of Tubulin, Actin and Gapdh levels in young and aged heart tissue. **(c)** Quantitation of protein bands in western blot (n=3, ns = not significant).

Figure S3

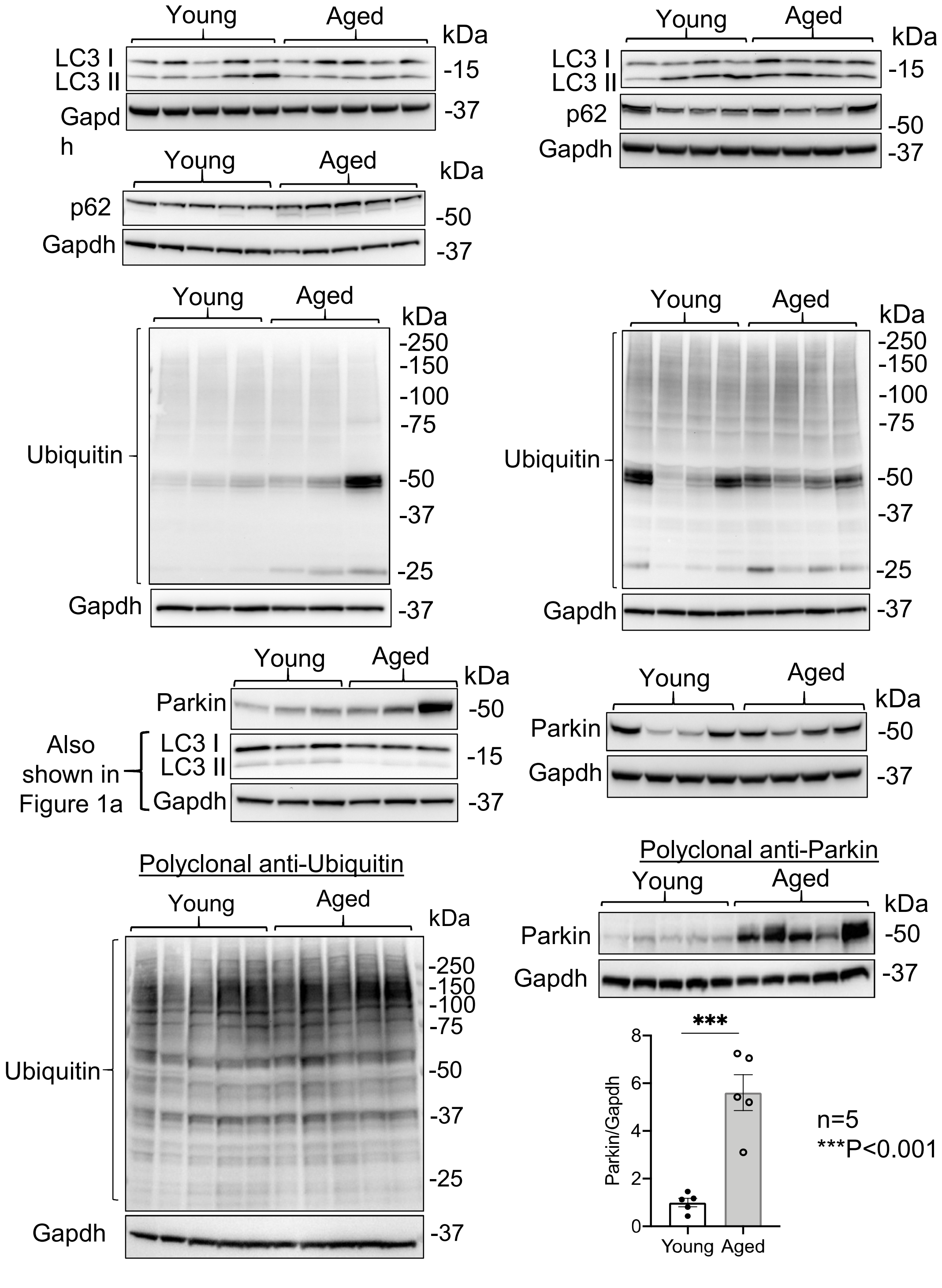

Figure S3. Additional Western blots for data shown in Figure 1a, 1b, 1d, 1e and 1f. LC3, p62, ubiquitin and Parkin levels in heart lysates of young and aged mice.

Figure S4

Loading order:

1. MW markers
2. Young heart1
3. Young heart 2
4. Young heart 3
5. Aged heart 1
6. Aged heart 2
7. Aged heart 3
8. MW markers
9. Young heart1
10. Aged heart2

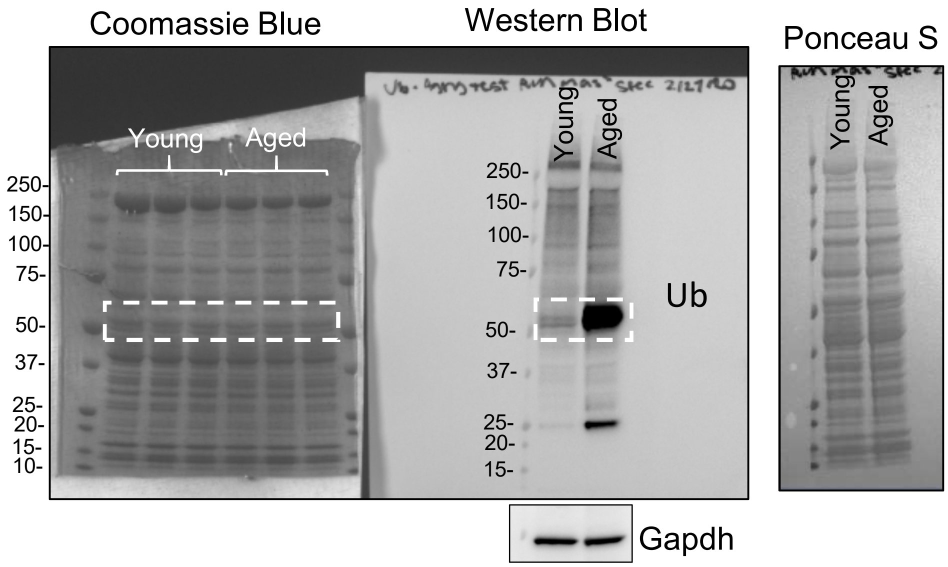

Figure S4. Proteins from young and aged hearts were separated on SDS-PAGE and the gel was cut at lane 8 (MW marker). The gel containing lanes 1-7 was stained with Commassie Blue for subsequent proteomics analysis and the gel containing lanes 9 and 10 was transferred to nitrocellulose membrane for immunoblotting blotting to confirm the position of the ubiquitin band(s). The area marked in white on Coomassie Blue stained gel corresponds to bands that were excised and analyzed by LC-MS/MS.

Figure S5

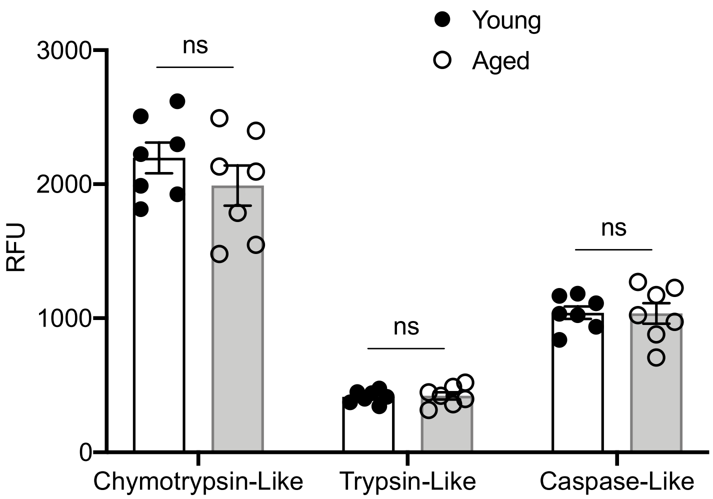

Figure S5. Proteasomal activities in young (4-month) and aged (24-month) hearts (n=7). Data represent mean ± SEM (ns = not significant).

Figure S6

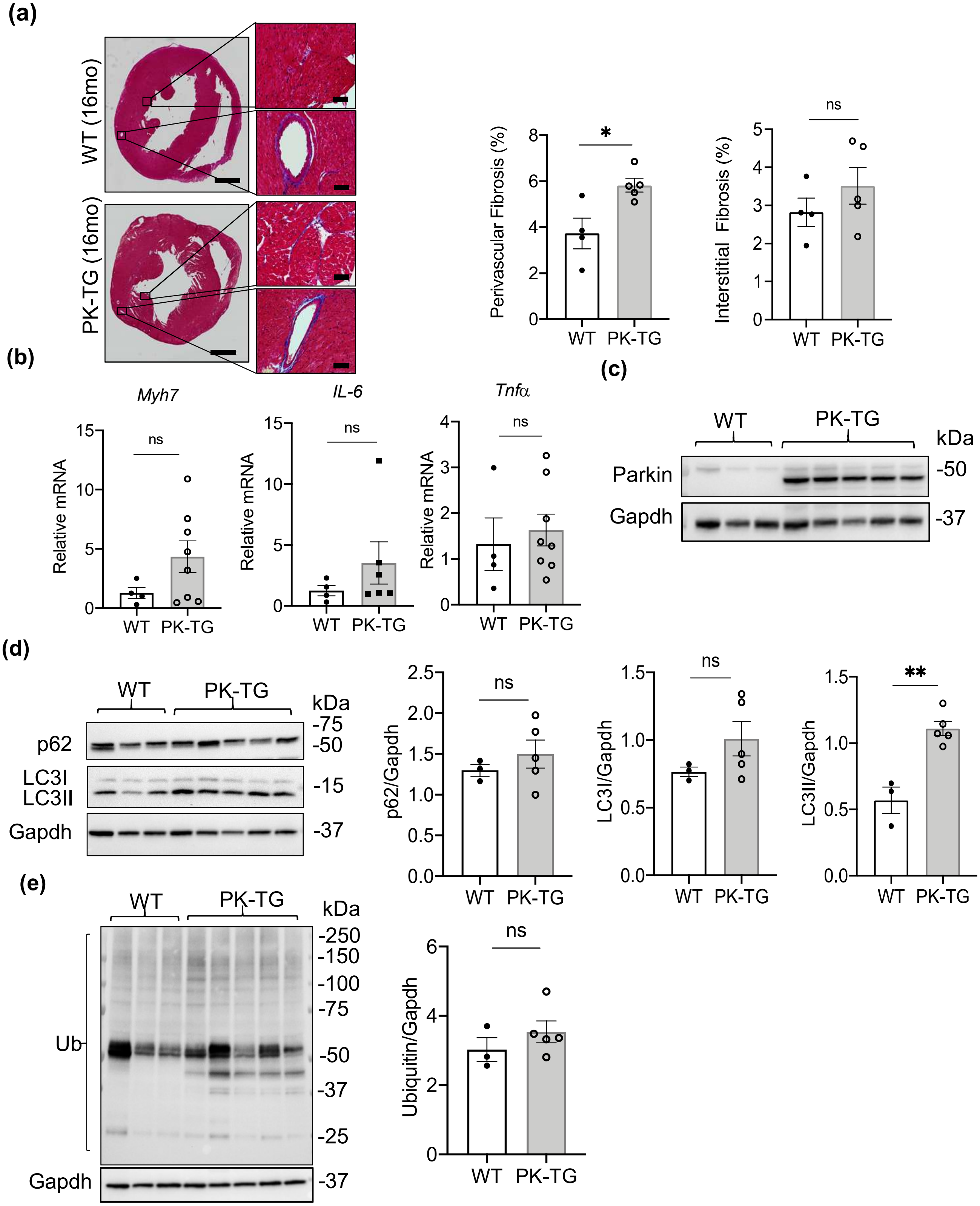

(a)
WT (16mo)
PK-TG (16mo)
Perivascular Fibrosis (%)
*
Interstitial Fibrosis (%)
ns
WT
PK-TG
(b)
Myh7
IL-6
Tnfα
Relative mRNA
ns
(c)
WT
PK-TG
kDa
Parkin
Gapdh
-50
-37
(d)
p62
LC3I
LC3II
Gapdh
-75
-50
-15
-37
p62/Gapdh
LC3I/Gapdh
LC3II/Gapdh
**
(e)
Ub
-250
-150
-100
-75
-50
-37
-25
Ubiquitin/Gapdh

Figure S6 cont.

Figure S6. Overexpression of Parkin in 16-month old mouse hearts leads to increased autophagic flux. **(a)** Masson's Trichrome staining and quantitation of perivascular and interstitial fibrosis in WT and Parkin transgenic (PK-TG) hearts at 16 months of age (n=4-5). **(b)** Analysis of *Myh7*, *IL-6* and *Tnfα* mRNA levels by qPCR in WT and Parkin TG (hearts (n=4-8). **(c)** Western blot for Parkin levels in hearts from WT and Parkin TG mice at 16 months of age. **(d)** Representative Western blots for LC3I, LC3II, and p62 levels. **(e)** Quantitation of protein levels in 16-month old WT and Parkin TG mouse hearts (n=3-5). **(f)** Representative blot and quantitation of total ubiquitinated protein levels in 16 month of WT and Parkin transgenic mouse hearts using a monoclonal ubiquitin antibody (n=3-5). Data are mean ± SEM (*P <0.05, **P<0.01, ns = not significant).

Figure S7

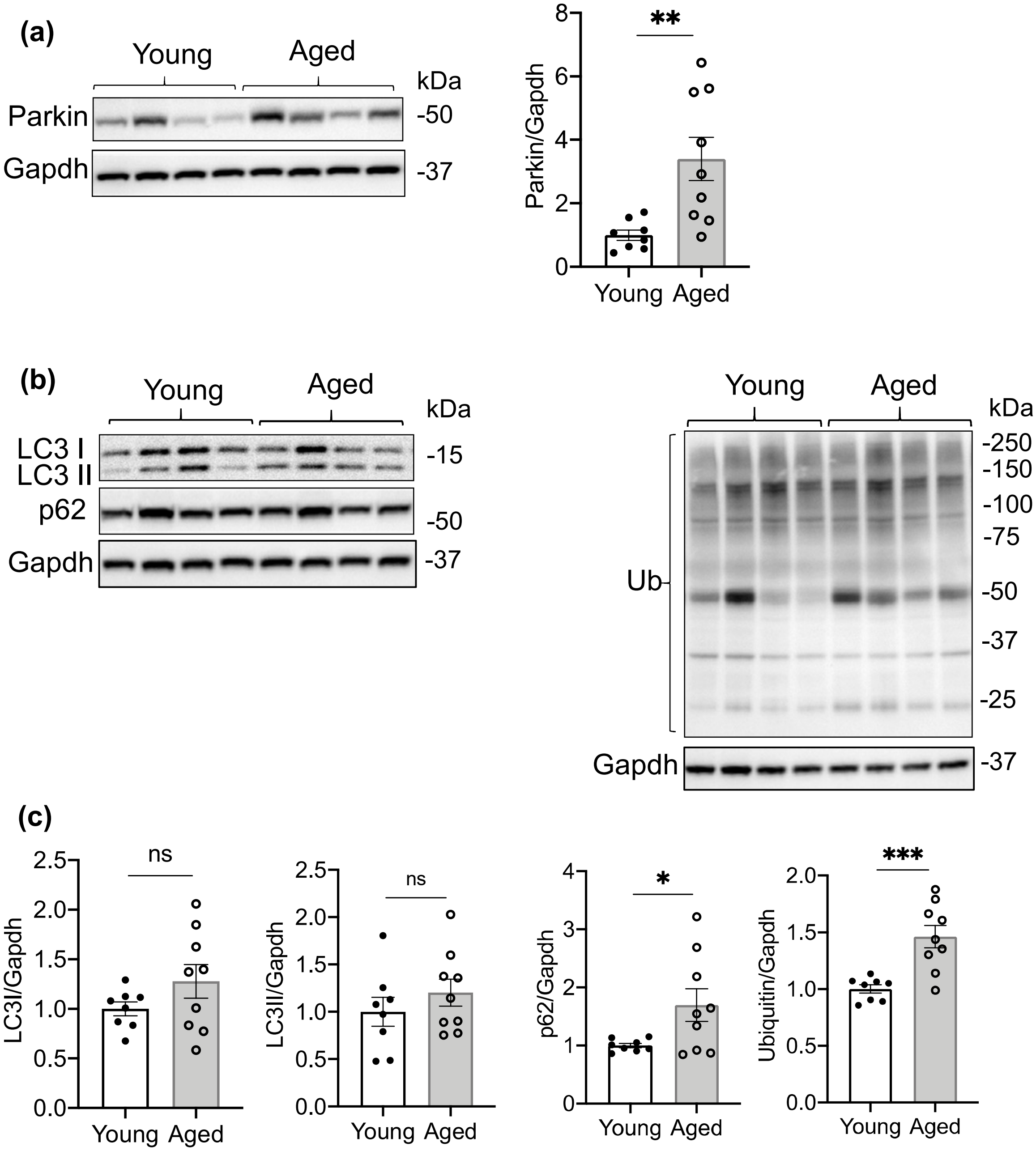

Figure S7. Autophagic activity in young and aged liver tissue. Representative Western blots of (**a**), Parkin, and (**b**) LC3, p62 and total ubiquitination protein levels in young and aged liver tissue. A monoclonal ubiquitin antibody was used for the Western blots. (**c**) Quantitation of protein levels (n=8-9). Data are mean $\pm$ SEM (*P < 0.05, **P <0.01, ***P<0.001, ns = not significant).

Figure S8

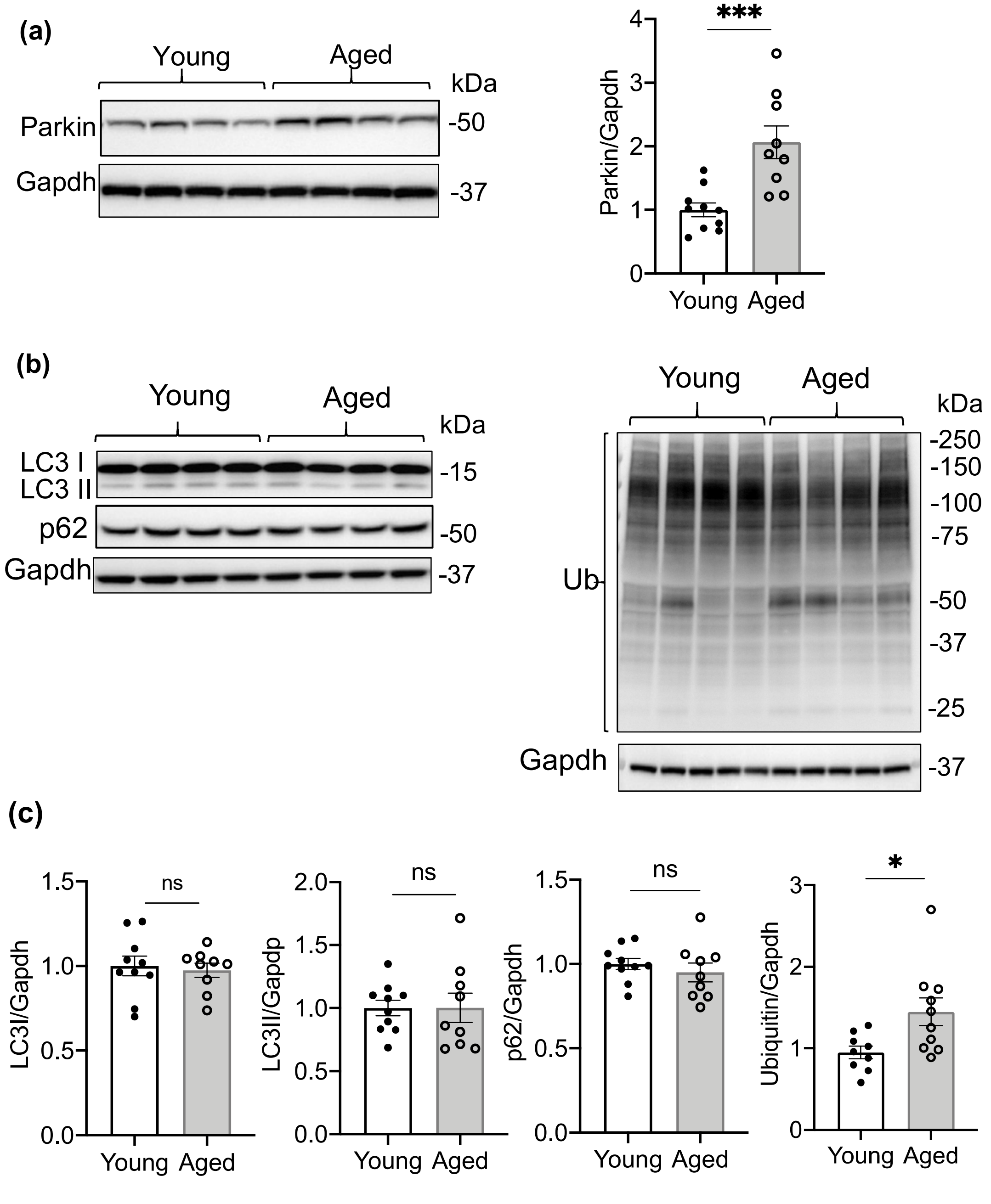

Figure S8 Autophagic activity in young and aged brain tissue. Representative Western blots of (**a**), Parkin, and (**b**) LC3, p62 and total ubiquitination protein levels in young and aged brain tissue. A monoclonal ubiquitin antibody was used for the Western blots. (**c**) Quantitation of protein levels (n=9-10). Data are mean ± SEM (*P < 0.05, ***P<0.001, ns = not significant).

Figure S9

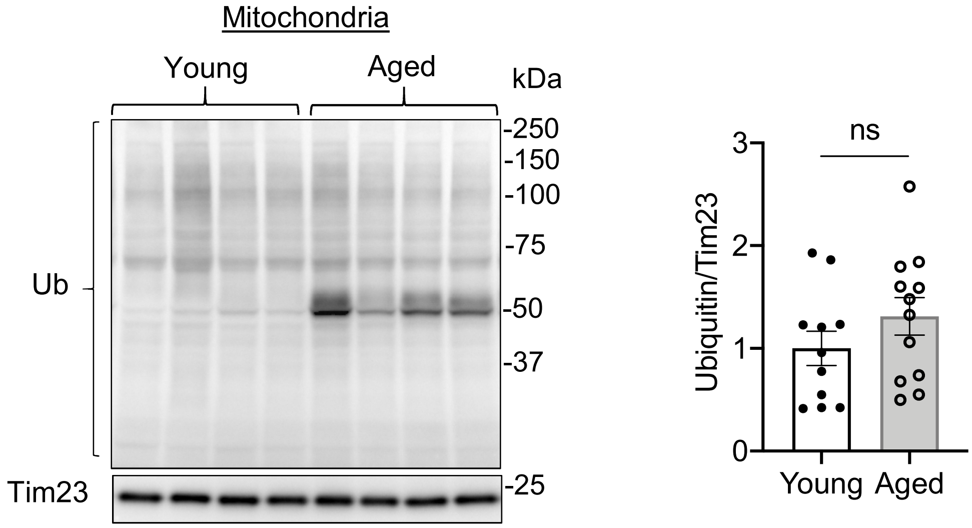

Figure S9. Representative Western blot and quantitation of ubiquitin levels in the mitochondrial fraction from young and aged hearts using a monoclonal anti-ubiquitin (n=11-12, ns = not significant).

Supplemental Table 1--- Young heart #1-identities of peptides with a minimum of 40% coverage

Protein Description	Accession	Coverage (%)	Avg. Mass (MW)
**ATP synthase subunit alpha**	Q03265|ATPA_MOUSE	82	59753
**ATP synthase subunit beta**	P56480|ATPB_MOUSE	91	56301
**60 kDa heat shock protein**	P63038|CH60_MOUSE	78	60956
**Serum albumin**	P07724|ALBU_MOUSE	80	68693
**Pyruvate kinase**	P52480|KPYM_MOUSE	71	57845
**Trifunctional enzyme subunit beta**	Q99JY0|ECHB_MOUSE	78	51386
**Beta-enolase**	P21550|ENOB_MOUSE	80	47025
**Dihydrolipoyl dehydrogenase**	O08749|DLDH_MOUSE	78	54272
**Sarcalumenin**	Q7TQ48|SRCA_MOUSE	40	99184
**Electron transfer flavoprotein-ubiquinone oxidoreductase**	Q921G7|ETFD_MOUSE	70	68091
**Fumarate hydratase**	P97807|FUMH_MOUSE	76	54357
**Selenium-binding protein 1**	P17563|SBP1_MOUSE	79	52514
**Tubulin beta-4B chain**	P68372|TBB4B_MOUSE	79	49831
**Aldehyde dehydrogenase**	P47738|ALDH2_MOUSE	59	56538
**Very long-chain specific acyl-CoA dehydrogenase**	P50544|ACADV_MOUSE	54	70876
**Phosphoglucomutase-1**	Q9D0F9|PGM1_MOUSE	76	61418
**Tubulin beta-5 chain**	P99024|TBB5_MOUSE	74	49671
**Tubulin beta-2A chain**	Q7TMM9|TBB2A_MOUSE	78	49907
**Alpha-1-antitrypsin 1-3**	Q00896|A1AT3_MOUSE	59	45823
**Tubulin beta-2B chain**	Q9CWF2|TBB2B_MOUSE	79	49953
**Alpha-1-antitrypsin 1-1**	P07758|A1AT1_MOUSE	59	46003
**Isocitrate dehydrogenase [NADP]**	P54071|IDHP_MOUSE	58	50906
**Glucose-6-phosphate isomerase**	P06745|G6PI_MOUSE	63	62767
**Alpha-1-antitrypsin 1-2**	P22599|A1AT2_MOUSE	61	45975
**Methylmalonate-semialdehyde dehydrogenase [acylating]**	Q9EQ20|MMSA_MOUSE	58	57916
**Alpha-1-antitrypsin 1-4**	Q00897|A1AT4_MOUSE	67	45998
**Dihydrolipoyllysine-residue acetyltransferase component of pyruvate dehydrogenase complex**	Q8BMF4|ODP2_MOUSE	57	67942
**Alpha-enolase**	P17182|ENOA_MOUSE	80	47141
**Succinyl-CoA:3-ketoacid coenzyme A transferase 1**	Q9D0K2|SCOT1_MOUSE	70	55989
**Selenium-binding protein 2**	Q63836|SBP2_MOUSE	65	52610
**Elongation factor Tu**	Q8BFR5|EFTU_MOUSE	68	49508
**EH domain-containing protein 2**	Q8BH64|EHD2_MOUSE	68	61175
**EH domain-containing protein 1**	Q9WVK4|EHD1_MOUSE	62	60603
**Tubulin beta-4A chain**	Q9D6F9|TBB4A_MOUSE	66	49586
**Elongation factor 1-alpha 2**	P62631|EF1A2_MOUSE	74	50454
**Tubulin beta-3 chain**	Q9ERD7|TBB3_MOUSE	47	50419
**Apoptosis-inducing factor 1**	Q9Z0X1|AIFM1_MOUSE	54	66766
**Tripartite motif-containing protein 72**	Q1XH17|TRI72_MOUSE	70	52817
**Alpha-1-antitrypsin 1-5**	Q00898|A1AT5_MOUSE	53	45891
**Bifunctional epoxide hydrolase 2**	P34914|HYES_MOUSE	68	62515
**Methylcrotonoyl-CoA carboxylase beta chain**	Q3ULD5|MCCB_MOUSE	54	61379
**Propionyl-CoA carboxylase beta chain**	Q99MN9|PCCB_MOUSE	65	58409
**Tubulin alpha-1B chain**	P05213|TBA1B_MOUSE	61	50152
**EH domain-containing protein 4**	Q9EQP2|EHD4_MOUSE	56	61481
**Tubulin alpha-1A chain**	P68369|TBA1A_MOUSE	60	50136
**Protein disulfide-isomerase A3**	P27773|PDIA3_MOUSE	57	56678
**Tubulin alpha-4A chain**	P68368|TBA4A_MOUSE	61	49924
**Glutamate dehydrogenase 1**	P26443|DHE3_MOUSE	63	61337
**Tubulin alpha-1C chain**	P68373|TBA1C_MOUSE	55	49909
**Acyl-CoA dehydrogenase family member 9**	Q8JZN5|ACAD9_MOUSE	52	68722
**NADH dehydrogenase [ubiquinone] flavoprotein 1**	Q91YT0|NDUV1_MOUSE	60	50834
**Carnitine O-acetyltransferase**	P47934|CACP_MOUSE	41	70840
**Delta-1-pyrroline-5-carboxylate dehydrogenase**	Q8CHT0|AL4A1_MOUSE	62	61841
**Serine/threonine-protein phosphatase 2A 65 kDa regulatory subunit A alpha isoform**	Q76MZ3|2AAA_MOUSE	56	65323

**Vimentin**	P20152|VIME_MOUSE	59	53688
**Calsequestrin-2**	O09161|CASQ2_MOUSE	45	48176
**Hydroxysteroid dehydrogenase-like protein 2**	Q2TPA8|HSDL2_MOUSE	61	54208
**Cytochrome b-c1 complex subunit 1**	Q9CZ13|QCR1_MOUSE	58	52852
**Dihydrolipoyllysine-residue succinyltransferase component of 2-oxoglutarate dehydrogenase complex**	Q9D2G2|ODO2_MOUSE	50	48995
**Protein disulfide-isomerase**	P09103|PDIA1_MOUSE	53	57059
**Elongation factor 1-alpha 1**	P10126|EF1A1_MOUSE	54	50114
**Tubulin alpha-3 chain**	P05214|TBA3_MOUSE	50	49960
**Tubulin alpha-8 chain**	Q9JJZ2|TBA8_MOUSE	50	50052
**Rab GDP dissociation inhibitor beta**	Q61598|GDIB_MOUSE	68	50537
**T-complex protein 1 subunit theta**	P42932|TCPQ_MOUSE	47	59556
**Atypical kinase ADCK3**	Q60936|ADCK3_MOUSE	46	71743
**Tubulin beta-6 chain**	Q922F4|TBB6_MOUSE	47	50090
**Pyruvate dehydrogenase protein X component**	Q8BKZ9|ODPX_MOUSE	56	53999
**Carboxylesterase 1D**	Q8VCT4|CES1D_MOUSE	50	61788
**Catalase**	P24270|CATA_MOUSE	44	59795
**Dihydropyrimidinase-related protein 2**	O08553|DPYL2_MOUSE	52	62278
**UPF0317 protein C14orf159 homolog**	Q8BH86|CN159_MOUSE	45	66366
**WD repeat-containing protein 1**	O88342|WDR1_MOUSE	48	66407
**T-complex protein 1 subunit alpha**	P11983|TCPA_MOUSE	53	60449
**Histone-lysine N-methyltransferase Smyd1**	P97443|SMYD1_MOUSE	53	56496
**NADP-dependent malic enzyme**	Q8BMF3|MAON_MOUSE	54	67098
**Aspartate--tRNA ligase cytoplasmic**	Q922B2|SYDC_MOUSE	51	57147
**Nicotinamide phosphoribosyltransferase**	Q99KQ4|NAMPT_MOUSE	50	55447
**Elongation factor 1-gamma**	Q9D8N0|EF1G_MOUSE	40	50061
**Lipoamide acyltransferase component of branched-chain alpha-keto acid dehydrogenase complex**	P53395|ODB2_MOUSE	48	53247
**Succinate-semialdehyde dehydrogenase**	Q8BWF0|SSDH_MOUSE	53	55968
**UTP--glucose-1-phosphate uridylyltransferase**	Q91ZJ5|UGPA_MOUSE	60	56979
**Fibrinogen beta chain**	Q8K0E8|FIBB_MOUSE	44	54753
**Cytosol aminopeptidase**	Q9CPY7|AMPL_MOUSE	48	56141
**Polymerase I and transcript release factor**	O54724|PTRF_MOUSE	43	43954
**Heterogeneous nuclear ribonucleoprotein K**	P61979|HNRPK_MOUSE	48	50976
**T-complex protein 1 subunit delta**	P80315|TCPD_MOUSE	47	58066
**Sorting and assembly machinery component 50 homolog**	Q8BGH2|SAM50_MOUSE	53	51864
**Perilipin-3**	Q9DBG5|PLIN3_MOUSE	44	47262
**ADP/ATP translocase 1**	P48962|ADT1_MOUSE	42	32904
**Adenylosuccinate synthetase isozyme 1**	P28650|PURA1_MOUSE	41	50254
**Retinal dehydrogenase 1**	P24549|AL1A1_MOUSE	43	54468
**Rab GDP dissociation inhibitor alpha**	P50396|GDIA_MOUSE	44	50522
**Malonyl-CoA decarboxylase**	Q99J39|DCMC_MOUSE	42	54736

Supplemental Table 2--- Young heart #2-identities of peptides with a minimum of 40% coverage

Protein Description	Accession	Coverage (%)	Avg. Mass (MW)
**ATP synthase subunit alpha**	Q03265|ATPA_MOUSE	78	59753
**ATP synthase subunit beta**	P56480|ATPB_MOUSE	89	56301
**Serum albumin**	P07724|ALBU_MOUSE	84	68693
**60 kDa heat shock protein**	P63038|CH60_MOUSE	79	60956
**Very long-chain specific acyl-CoA dehydrogenase**	P50544|ACADV_MOUSE	76	70876
**Pyruvate kinase**	P52480|KPYM_MOUSE	76	57845
**Sarcalumenin**	Q7TQ48|SRCA_MOUSE	40	99184
**Trifunctional enzyme subunit beta**	Q99JY0|ECHB_MOUSE	76	51386
**Electron transfer flavoprotein-ubiquinone oxidoreductase**	Q921G7|ETFD_MOUSE	68	68091
**Beta-enolase**	P21550|ENOB_MOUSE	77	47025
**Dihydrolipoyl dehydrogenase**	O08749|DLDH_MOUSE	70	54272
**Fumarate hydratase**	P97807|FUMH_MOUSE	81	54357
**Phosphoglucomutase-1**	Q9D0F9|PGM1_MOUSE	84	61418
**Selenium-binding protein 1**	P17563|SBP1_MOUSE	74	52514
**Dihydrolipoyllysine-residue acetyltransferase component of pyruvate dehydrogenase complex**	Q8BMF4|ODP2_MOUSE	63	67942
**Tubulin beta-4B chain**	P68372|TBB4B_MOUSE	72	49831
**Glucose-6-phosphate isomerase**	P06745|G6PI_MOUSE	62	62767
**Isocitrate dehydrogenase [NADP]**	P54071|IDHP_MOUSE	56	50906
**Alpha-1-antitrypsin 1-3**	Q00896|A1AT3_MOUSE	57	45823
**Aldehyde dehydrogenase**	P47738|ALDH2_MOUSE	68	56538
**Alpha-1-antitrypsin 1-1**	P07758|A1AT1_MOUSE	55	46003
**Alpha-enolase**	P17182|ENOA_MOUSE	73	47141
**Tubulin beta-5 chain**	P99024|TBB5_MOUSE	71	49671
**Alpha-1-antitrypsin 1-2**	P22599|A1AT2_MOUSE	57	45975
**Tubulin beta-2A chain**	Q7TMM9|TBB2A_MOUSE	72	49907
**EH domain-containing protein 2**	Q8BH64|EHD2_MOUSE	71	61175
**Alpha-1-antitrypsin 1-4**	Q00897|A1AT4_MOUSE	63	45998
**Selenium-binding protein 2**	Q63836|SBP2_MOUSE	67	52610
**Succinyl-CoA:3-ketoacid coenzyme A transferase 1**	Q9D0K2|SCOT1_MOUSE	62	55989
**Methylmalonate-semialdehyde dehydrogenase [acylating]**	Q9EQ20|MMSA_MOUSE	66	57916
**Alpha-1-antitrypsin 1-5**	Q00898|A1AT5_MOUSE	55	45891
**EH domain-containing protein 1**	Q9WVK4|EHD1_MOUSE	62	60603
**Tripartite motif-containing protein 72**	Q1XH17|TRI72_MOUSE	74	52817
**Tubulin beta-4A chain**	Q9D6F9|TBB4A_MOUSE	65	49586
**Tubulin alpha-1A chain**	P68369|TBA1A_MOUSE	63	50136
**Apoptosis-inducing factor 1**	Q9Z0X1|AIFM1_MOUSE	53	66766
**Tubulin alpha-1B chain**	P05213|TBA1B_MOUSE	63	50152
**Bifunctional epoxide hydrolase 2**	P34914|HYES_MOUSE	68	62515
**Elongation factor Tu**	Q8BFR5|EFTU_MOUSE	62	49508
**Tubulin alpha-4A chain**	P68368|TBA4A_MOUSE	62	49924
**EH domain-containing protein 4**	Q9EQP2|EHD4_MOUSE	60	61481
**Elongation factor Tu**	tr|D3YVN7|D3YVN7_MOUSE	62	49538
**Tubulin beta-3 chain**	Q9ERD7|TBB3_MOUSE	51	50419
**Tubulin alpha-1C chain**	P68373|TBA1C_MOUSE	58	49909
**Elongation factor 1-alpha 2**	P62631|EF1A2_MOUSE	60	50454
**Propionyl-CoA carboxylase beta chain**	Q99MN9|PCCB_MOUSE	56	58409
**Dihydrolipoyllysine-residue succinyltransferase component of 2-oxoglutarate dehydrogenase complex**	Q9D2G2|ODO2_MOUSE	61	48995
**Protein disulfide-isomerase A3**	P27773|PDIA3_MOUSE	59	56678
**NADH dehydrogenase [ubiquinone] flavoprotein 1**	Q91YT0|NDUV1_MOUSE	64	50834
**Acyl-CoA dehydrogenase family member 9**	Q8JZN5|ACAD9_MOUSE	54	68722
**Methylcrotonoyl-CoA carboxylase beta chain**	Q3ULD5|MCCB_MOUSE	53	61379
**Vimentin**	P20152|VIME_MOUSE	64	53688
**Glutamate dehydrogenase 1**	P26443|DHE3_MOUSE	56	61337
**Calsequestrin-2**	O09161|CASQ2_MOUSE	44	48176

**Delta-1-pyrroline-5-carboxylate dehydrogenase**	Q8CHT0|AL4A1_MOUSE	57	61841
**Cytochrome b-c1 complex subunit 1**	Q9CZ13|QCR1_MOUSE	59	52852
**Tubulin alpha-3 chain**	P05214|TBA3_MOUSE	52	49960
**Carnitine O-acetyltransferase**	P47934|CACP_MOUSE	41	70840
**Rab GDP dissociation inhibitor beta**	Q61598|GDIB_MOUSE	65	50537
**Tubulin alpha-8 chain**	Q9JJZ2|TBA8_MOUSE	50	50052
**Catalase**	P24270|CATA_MOUSE	51	59795
**Hydroxysteroid dehydrogenase-like protein 2**	Q2TPA8|HSDL2_MOUSE	61	54208
**Dihydropyrimidinase-related protein 2**	O08553|DPYL2_MOUSE	66	62278
**Malonyl-CoA decarboxylase mitochondrial**	Q99J39|DCMC_MOUSE	55	54736
**WD repeat-containing protein 1**	O88342|WDR1_MOUSE	49	66407
**Atypical kinase ADCK3 mitochondrial**	Q60936|ADCK3_MOUSE	43	71743
**Elongation factor 1-alpha 1**	P10126|EF1A1_MOUSE	46	50114
**Protein disulfide-isomerase**	P09103|PDIA1_MOUSE	52	57059
**NADP-dependent malic enzyme mitochondrial**	Q8BMF3|MAON_MOUSE	48	67098
**T-complex protein 1 subunit theta**	P42932|TCPQ_MOUSE	43	59556
**Tubulin beta-6 chain**	Q922F4|TBB6_MOUSE	42	50090
**Serine/threonine-protein phosphatase 2A 65 kDa regulatory subunit A alpha isoform**	Q76MZ3|2AAA_MOUSE	52	65323
**UPF0317 protein C14orf159 homolog**	Q8BH86|CN159_MOUSE	48	66366
**Aspartate--tRNA ligase cytoplasmic**	Q922B2|SYDC_MOUSE	52	57147
**Nicotinamide phosphoribosyltransferase**	Q99KQ4|NAMPT_MOUSE	50	55447
**Pyruvate dehydrogenase protein X component**	Q8BKZ9|ODPX_MOUSE	58	53999
**Polymerase I and transcript release factor**	O54724|PTRF_MOUSE	49	43954
**UTP--glucose-1-phosphate uridylyltransferase**	Q91ZJ5|UGPA_MOUSE	46	56979
**Lipoamide acyltransferase component of branched-chain alpha-keto acid dehydrogenase complex**	P53395|ODB2_MOUSE	64	53247
**Bifunctional purine biosynthesis protein**	Q9CWJ9|PUR9_MOUSE	46	64217
**Elongation factor 1-gamma**	Q9D8N0|EF1G_MOUSE	43	50061
**Fibrinogen beta chain**	Q8K0E8|FIBB_MOUSE	41	54753
**Histone-lysine N-methyltransferase Smyd1**	P97443|SMYD1_MOUSE	54	56496
**T-complex protein 1 subunit alpha**	P11983|TCPA_MOUSE	45	60449
**T-complex protein 1 subunit beta**	P80314|TCPB_MOUSE	56	57477
**Carboxylesterase 1D**	Q8VCT4|CES1D_MOUSE	55	61788
**Retinal dehydrogenase 1**	P24549|AL1A1_MOUSE	63	54468
**Desmin**	P31001|DESM_MOUSE	46	53498
**Heterogeneous nuclear ribonucleoprotein K**	P61979|HNRPK_MOUSE	41	50976
**Perilipin-3**	Q9DBG5|PLIN3_MOUSE	44	47262
**Sorting and assembly machinery component 50 homolog**	Q8BGH2|SAM50_MOUSE	42	51864
**Rab GDP dissociation inhibitor alpha**	P50396|GDIA_MOUSE	49	50522
**Adenylosuccinate synthetase isozyme 1**	P28650|PURA1_MOUSE	41	50254
**Actin alpha cardiac muscle 1 OS=Mus musculus GN=Actc1 PE=1 SV=1**	P68033|ACTC_MOUSE	45	42019

Supplemental Table 3--- Young #3-identities of peptides with a minimum of 40% coverage

Protein Description	Accession	Coverage (%)	Avg. Mass (MW)
**ATP synthase subunit beta**	P56480|ATPB_MOUSE	92	56301
**ATP synthase subunit alpha**	Q03265|ATPA_MOUSE	83	59753
**Serum albumin**	P07724|ALBU_MOUSE	86	68693
**60 kDa heat shock protein**	P63038|CH60_MOUSE	76	60956
**Very long-chain specific acyl-CoA dehydrogenase**	P50544|ACADV_MOUSE	78	70876
**Beta-enolase**	P21550|ENOB_MOUSE	80	47025
**Trifunctional enzyme subunit beta**	Q99JY0|ECHB_MOUSE	76	51386
**Dihydrolipoyl dehydrogenase**	O08749|DLDH_MOUSE	78	54272
**Pyruvate kinase**	P52480|KPYM_MOUSE	76	57845
**Sarcalumenin**	Q7TQ48|SRCA_MOUSE	40	99184
**Electron transfer flavoprotein-ubiquinone oxidoreductase**	Q921G7|ETFD_MOUSE	74	68091
**Alpha-enolase**	P17182|ENOA_MOUSE	84	47141
**Phosphoglucomutase-1**	Q9D0F9|PGM1_MOUSE	80	61418
**Fumarate hydratase**	P97807|FUMH_MOUSE	75	54357
**Isocitrate dehydrogenase [NADP]**	P54071|IDHP_MOUSE	60	50906
**Glucose-6-phosphate isomerase**	P06745|G6PI_MOUSE	61	62767
**Dihydrolipoyllysine-residue acetyltransferase component of pyruvate dehydrogenase complex**	Q8BMF4|ODP2_MOUSE	61	67942
**Selenium-binding protein 1**	P17563|SBP1_MOUSE	81	52514
**Aldehyde dehydrogenase**	P47738|ALDH2_MOUSE	67	56538
**Succinyl-CoA:3-ketoacid coenzyme A transferase 1**	Q9D0K2|SCOT1_MOUSE	70	55989
**Tubulin beta-4B chain**	P68372|TBB4B_MOUSE	71	49831
**Elongation factor 1-alpha 2**	P62631|EF1A2_MOUSE	74	50454
**EH domain-containing protein 2**	Q8BH64|EHD2_MOUSE	70	61175
**Tubulin beta-5 chain**	P99024|TBB5_MOUSE	68	49671
**Methylmalonate-semialdehyde dehydrogenase [acylating]**	Q9EQ20|MMSA_MOUSE	71	57916
**Alpha-1-antitrypsin 1-2**	P22599|A1AT2_MOUSE	67	45975
**Tubulin beta-2A chain**	Q7TMM9|TBB2A_MOUSE	65	49907
**Alpha-1-antitrypsin 1-4**	Q00897|A1AT4_MOUSE	66	45998
**Selenium-binding protein 2**	Q63836|SBP2_MOUSE	72	52610
**Tubulin beta-2B chain**	Q9CWF2|TBB2B_MOUSE	62	49953
**Apoptosis-inducing factor 1**	Q9Z0X1|AIFM1_MOUSE	56	66766
**Alpha-1-antitrypsin 1-3**	Q00896|A1AT3_MOUSE	55	45823
**Alpha-1-antitrypsin 1-1**	P07758|A1AT1_MOUSE	52	46003
**Bifunctional epoxide hydrolase 2**	P34914|HYES_MOUSE	67	62515
**Propionyl-CoA carboxylase beta chain**	Q99MN9|PCCB_MOUSE	59	58409
**EH domain-containing protein 1**	Q9WVK4|EHD1_MOUSE	58	60603
**Tripartite motif-containing protein 72**	Q1XH17|TRI72_MOUSE	77	52817
**Elongation factor Tu**	Q8BFR5|EFTU_MOUSE	62	49508
**EH domain-containing protein 4**	Q9EQP2|EHD4_MOUSE	57	61481
**Glutamate dehydrogenase 1**	P26443|DHE3_MOUSE	61	61337
**Tubulin beta-3 chain**	Q9ERD7|TBB3_MOUSE	43	50419
**Alpha-1-antitrypsin 1-5**	Q00898|A1AT5_MOUSE	50	45891
**Carnitine O-acetyltransferase**	P47934|CACP_MOUSE	43	70840
**Cytochrome b-c1 complex subunit 1**	Q9CZ13|QCR1_MOUSE	50	52852
**Vimentin**	P20152|VIME_MOUSE	65	53688
**Tubulin beta-4A chain**	Q9D6F9|TBB4A_MOUSE	59	49586
**Hydroxysteroid dehydrogenase-like protein 2**	Q2TPA8|HSDL2_MOUSE	65	54208
**Protein disulfide-isomerase A3**	P27773|PDIA3_MOUSE	60	56678
**Methylcrotonoyl-CoA carboxylase beta chain**	Q3ULD5|MCCB_MOUSE	51	61379
**NADH dehydrogenase [ubiquinone] flavoprotein 1**	Q91YT0|NDUV1_MOUSE	62	50834
**Dihydrolipoyllysine-residue succinyltransferase component of 2-oxoglutarate dehydrogenase complex**	Q9D2G2|ODO2_MOUSE	58	48995
**Serine/threonine-protein phosphatase 2A 65 kDa regulatory subunit A alpha isoform**	Q76MZ3|2AAA_MOUSE	56	65323
**Catalase**	P24270|CATA_MOUSE	51	59795

**Tubulin alpha-1A chain**	P68369|TBA1A_MOUSE	62	50136
**Tubulin alpha-1B chain**	P05213|TBA1B_MOUSE	62	50152
**Carboxylesterase 1D**	Q8VCT4|CES1D_MOUSE	56	61788
**Tubulin alpha-4A chain**	P68368|TBA4A_MOUSE	61	49924
**Acyl-CoA dehydrogenase family member 9**	Q8JZN5|ACAD9_MOUSE	55	68722
**Aspartate--tRNA ligase cytoplasmic**	Q922B2|SYDC_MOUSE	57	57147
**Dihydropyrimidinase-related protein 2**	O08553|DPYL2_MOUSE	60	62278
**Elongation factor 1-alpha 1**	P10126|EF1A1_MOUSE	51	50114
**Tubulin alpha-1C chain**	P68373|TBA1C_MOUSE	57	49909
**Calsequestrin-2**	O09161|CASQ2_MOUSE	45	48176
**Protein disulfide-isomerase**	P09103|PDIA1_MOUSE	51	57059
**NADP-dependent malic enzyme**	Q8BMF3|MAON_MOUSE	56	67098
**Delta-1-pyrroline-5-carboxylate dehydrogenase**	Q8CHT0|AL4A1_MOUSE	49	61841
**Serine protease inhibitor A3K**	P07759|SPA3K_MOUSE	45	46880
**Rab GDP dissociation inhibitor beta**	Q61598|GDIB_MOUSE	52	50537
**Amine oxidase [flavin-containing] B**	Q8BW75|AOFB_MOUSE	48	58558
**Heterogeneous nuclear ribonucleoprotein K**	P61979|HNRPK_MOUSE	48	50976
**Lipoamide acyltransferase component of branched-chain alpha-keto acid dehydrogenase complex**	P53395|ODB2_MOUSE	48	53247
**Tubulin alpha-8 chain**	Q9JJZ2|TBA8_MOUSE	48	50052
**Tubulin alpha-3 chain**	P05214|TBA3_MOUSE	50	49960
**Histone-lysine N-methyltransferase Smyd1**	P97443|SMYD1_MOUSE	54	56496
**Pyruvate dehydrogenase protein X component**	Q8BKZ9|ODPX_MOUSE	55	53999
**Polymerase I and transcript release factor**	O54724|PTRF_MOUSE	42	43954
**Nicotinamide phosphoribosyltransferase**	Q99KQ4|NAMPT_MOUSE	46	55447
**T-complex protein 1 subunit theta**	P42932|TCPQ_MOUSE	43	59556
**UTP--glucose-1-phosphate uridylyltransferase**	Q91ZJ5|UGPA_MOUSE	47	56979
**Fibrinogen beta chain**	Q8K0E8|FIBB_MOUSE	47	54753
**Leukotriene A-4 hydrolase**	P24527|LKHA4_MOUSE	49	69051
**Stress-induced-phosphoprotein 1**	Q60864|STIP1_MOUSE	41	62582
**Cytosol aminopeptidase**	Q9CPY7|AMPL_MOUSE	54	56141
**T-complex protein 1 subunit beta**	P80314|TCPB_MOUSE	48	57477
**Sorting and assembly machinery component 50**	Q8BGH2|SAM50_MOUSE	48	51864
**26S proteasome non-ATPase regulatory subunit 3**	P14685|PSMD3_MOUSE	43	60718
**Rab GDP dissociation inhibitor alpha**	P50396|GDIA_MOUSE	52	50522
**Actin alpha cardiac muscle 1**	P68033|ACTC_MOUSE	45	42019
**Calreticulin**	P14211|CALR_MOUSE	46	47995
**Malonyl-CoA decarboxylase**	Q99J39|DCMC_MOUSE	44	54736
**Perilipin-3**	Q9DBG5|PLIN3_MOUSE	56	47262
**Fibrinogen gamma chain**	Q8VCM7|FIBG_MOUSE	41	49391
**Neutral cholesterol ester hydrolase 1**	Q8BLF1|NCEH1_MOUSE	42	45740

Supplemental Table 4---Aged heart #1-identities of peptides with a minimum of 40% coverage

Protein Description	Accession	Coverage (%)	Avg. Mass (MW)
**ATP synthase subunit beta**	P56480|ATPB_MOUSE	89	56301
**ATP synthase subunit alpha**	Q03265|ATPA_MOUSE	90	59753
**Serum albumin**	P07724|ALBU_MOUSE	89	68693
**60 kDa heat shock protein**	P63038|CH60_MOUSE	81	60956
**Beta-enolase**	P21550|ENOB_MOUSE	78	47025
**Trifunctional enzyme subunit beta**	Q99JY0|ECHB_MOUSE	81	51386
**Dihydrolipoyl dehydrogenase**	O08749|DLDH_MOUSE	82	54272
**Pyruvate kinase**	P52480|KPYM_MOUSE	77	57845
**Electron transfer flavoprotein-ubiquinone oxidoreductase**	Q921G7|ETFD_MOUSE	73	68091
**Sarcalumenin**	Q7TQ48|SRCA_MOUSE	40	99184
**Selenium-binding protein 1**	P17563|SBP1_MOUSE	86	52514
**Very long-chain specific acyl-CoA dehydrogenase**	P50544|ACADV_MOUSE	74	70876
**Phosphoglucomutase-1**	Q9D0F9|PGM1_MOUSE	84	61418
**Isocitrate dehydrogenase [NADP]**	P54071|IDHP_MOUSE	71	50906
**Fumarate hydratase**	P97807|FUMH_MOUSE	75	54357
**Alpha-enolase**	P17182|ENOA_MOUSE	81	47141
**Aldehyde dehydrogenase**	P47738|ALDH2_MOUSE	76	56538
**Selenium-binding protein 2**	Q63836|SBP2_MOUSE	71	52610
**Succinyl-CoA:3-ketoacid coenzyme A transferase 1**	Q9D0K2|SCOT1_MOUSE	72	55989
**Tubulin beta-4B chain**	P68372|TBB4B_MOUSE	73	49831
**Glucose-6-phosphate isomerase**	P06745|G6PI_MOUSE	66	62767
**Tubulin beta-5 chain**	P99024|TBB5_MOUSE	70	49671
**Dihydrolipoyllysine-residue acetyltransferase component of pyruvate dehydrogenase complex**	Q8BMF4|ODP2_MOUSE	57	67942
**Tubulin beta-2A chain**	Q7TMM9|TBB2A_MOUSE	73	49907
**Elongation factor 1-alpha 2**	P62631|EF1A2_MOUSE	74	50454
**Tubulin beta-2B chain**	Q9CWF2|TBB2B_MOUSE	74	49953
**EH domain-containing protein 2**	Q8BH64|EHD2_MOUSE	76	61175
**Apoptosis-inducing factor 1**	Q9Z0X1|AIFM1_MOUSE	61	66766
**Methylmalonate-semialdehyde dehydrogenase [acylating]**	Q9EQ20|MMSA_MOUSE	65	57916
**Alpha-1-antitrypsin 1-4**	Q00897|A1AT4_MOUSE	63	45998
**Propionyl-CoA carboxylase beta chain**	Q99MN9|PCCB_MOUSE	67	58409
**EH domain-containing protein 1**	Q9WVK4|EHD1_MOUSE	68	60603
**Tripartite motif-containing protein 72**	Q1XH17|TRI72_MOUSE	75	52817
**Glutamate dehydrogenase 1**	P26443|DHE3_MOUSE	60	61337
**Cytochrome b-c1 complex subunit 1**	Q9CZ13|QCR1_MOUSE	71	52852
**Alpha-1-antitrypsin 1-3**	Q00896|A1AT3_MOUSE	55	45823
**Alpha-1-antitrypsin 1-1**	P07758|A1AT1_MOUSE	55	46003
**Elongation factor Tu**	Q8BFR5|EFTU_MOUSE	73	49508
**Vimentin**	P20152|VIME_MOUSE	65	53688
**Bifunctional epoxide hydrolase 2**	P34914|HYES_MOUSE	73	62515
**Alpha-1-antitrypsin 1-2**	P22599|A1AT2_MOUSE	55	45975
**EH domain-containing protein 4**	Q9EQP2|EHD4_MOUSE	64	61481
**Protein disulfide-isomerase A3**	P27773|PDIA3_MOUSE	63	56678
**NADH dehydrogenase [ubiquinone] flavoprotein 1**	Q91YT0|NDUV1_MOUSE	74	50834
**Tubulin beta-4A chain**	Q9D6F9|TBB4A_MOUSE	62	49586
**Rab GDP dissociation inhibitor beta**	Q61598|GDIB_MOUSE	71	50537
**Methylcrotonoyl-CoA carboxylase beta chain**	Q3ULD5|MCCB_MOUSE	51	61379
**Tubulin beta-3 chain**	Q9ERD7|TBB3_MOUSE	52	50419
**Tubulin alpha-1B chain**	P05213|TBA1B_MOUSE	62	50152
**Tubulin alpha-4A chain**	P68368|TBA4A_MOUSE	63	49924
**Dihydropyrimidinase-related protein 2**	O08553|DPYL2_MOUSE	71	62278
**Dihydrolipoyllysine-residue succinyltransferase component of 2-oxoglutarate dehydrogenase complex**	Q9D2G2|ODO2_MOUSE	50	48995
**Delta-1-pyrroline-5-carboxylate dehydrogenase**	Q8CHT0|AL4A1_MOUSE	54	61841
**Hydroxysteroid dehydrogenase-like protein 2**	Q2TPA8|HSDL2_MOUSE	67	54208

**Tubulin alpha-1A chain**	P68369|TBA1A_MOUSE	61	50136
**Catalase**	P24270|CATA_MOUSE	53	59795
**Alpha-1-antitrypsin 1-5**	Q00898|A1AT5_MOUSE	48	45891
**Serine/threonine-protein phosphatase 2A 65 kDa regulatory subunit A alpha isoform**	Q76MZ3|2AAA_MOUSE	57	65323
**Carnitine O-acetyltransferase**	P47934|CACP_MOUSE	44	70840
**Elongation factor 1-alpha 1**	P10126|EF1A1_MOUSE	65	50114
**Tubulin alpha-1C chain**	P68373|TBA1C_MOUSE	56	49909
**Fibrinogen beta chain**	Q8K0E8|FIBB_MOUSE	64	54753
**Amine oxidase [flavin-containing] B**	Q8BW75|AOFB_MOUSE	59	58558
**Protein disulfide-isomerase**	P09103|PDIA1_MOUSE	54	57059
**Carboxylesterase 1D**	Q8VCT4|CES1D_MOUSE	64	61788
**Calsequestrin-2**	O09161|CASQ2_MOUSE	50	48176
**Serine protease inhibitor A3K**	P07759|SPA3K_MOUSE	48	46880
**Acyl-CoA dehydrogenase family member 9**	Q8JZN5|ACAD9_MOUSE	49	68722
**WD repeat-containing protein 1**	O88342|WDR1_MOUSE	51	66407
**NADP-dependent malic enzyme**	Q8BMF3|MAON_MOUSE	46	67098
**Tubulin beta-6 chain**	Q922F4|TBB6_MOUSE	47	50090
**Cytosol aminopeptidase**	Q9CPY7|AMPL_MOUSE	71	56141
**Aspartate--tRNA ligase**	Q922B2|SYDC_MOUSE	58	57147
**Nicotinamide phosphoribosyltransferase**	Q99KQ4|NAMPT_MOUSE	52	55447
**UPF0317 protein C14orf159 homolog**	Q8BH86|CN159_MOUSE	54	66366
**Tubulin alpha-8 chain**	Q9JJZ2|TBA8_MOUSE	47	50052
**Aldehyde dehydrogenase X**	Q9CZS1|AL1B1_MOUSE	48	57553
**Tubulin alpha-3 chain**	P05214|TBA3_MOUSE	52	49960
**Stress-induced-phosphoprotein 1**	Q60864|STIP1_MOUSE	46	62582
**T-complex protein 1 subunit theta**	P42932|TCPQ_MOUSE	45	59556
**Vitamin D-binding protein**	P21614|VTDB_MOUSE	49	53600
**Histone-lysine N-methyltransferase Smyd1**	P97443|SMYD1_MOUSE	58	56496
**Actin alpha cardiac muscle 1**	P68033|ACTC_MOUSE	58	42019
**Pyruvate dehydrogenase protein X component**	Q8BKZ9|ODPX_MOUSE	66	53999
**Sorting and assembly machinery component 50 homolog**	Q8BGH2|SAM50_MOUSE	53	51864
**Bifunctional purine biosynthesis protein**	Q9CWJ9|PUR9_MOUSE	51	64217
**Antithrombin-III**	P32261|ANT3_MOUSE	63	52004
**UTP--glucose-1-phosphate uridylyltransferase**	Q91ZJ5|UGPA_MOUSE	55	56979
**Actin aortic smooth muscle**	P62737|ACTA_MOUSE	51	42009
**Succinate-semialdehyde dehydrogenase**	Q8BWF0|SSDH_MOUSE	44	55968
**Adenylyl cyclase-associated protein 1**	P40124|CAP1_MOUSE	59	51565
**Mitochondrial-processing peptidase subunit alpha**	Q9DC61|MPPA_MOUSE	43	58279

Supplemental Table 5 - Aged heart #2 - identities of peptides with a minimum of 40% coverage

Protein description	Accession	Coverage (%)	Avg. Mass (MW)
**ATP synthase subunit beta**	P56480|ATPB_MOUSE	89	56301
**ATP synthase subunit alpha**	Q03265|ATPA_MOUSE	82	59753
**Serum albumin**	P07724|ALBU_MOUSE	88	68693
**60 kDa heat shock protein**	P63038|CH60_MOUSE	79	60956
**Trifunctional enzyme subunit beta**	Q99JY0|ECHB_MOUSE	78	51386
**Beta-enolase**	P21550|ENOB_MOUSE	80	47025
**Pyruvate kinase**	P52480|KPYM_MOUSE	76	57845
**Isocitrate dehydrogenase [NADP]**	P54071|IDHP_MOUSE	74	50906
**Sarcalumenin**	Q7TQ48|SRCA_MOUSE	41	99184
**Dihydrolipoyl dehydrogenase**	O08749|DLDH_MOUSE	80	54272
**Alpha-enolase**	P17182|ENOA_MOUSE	81	47141
**Electron transfer flavoprotein-ubiquinone oxidoreductase**	Q921G7|ETFD_MOUSE	73	68091
**Phosphoglucomutase-1**	Q9D0F9|PGM1_MOUSE	79	61418
**Fumarate hydratase**	P97807|FUMH_MOUSE	77	54357
**Very long-chain specific acyl-CoA dehydrogenase**	P50544|ACADV_MOUSE	61	70876
**Selenium-binding protein 1**	P17563|SBP1_MOUSE	85	52514
**Aldehyde dehydrogenase**	P47738|ALDH2_MOUSE	72	56538
**Tubulin beta-4B chain**	P68372|TBB4B_MOUSE	77	49831
**Alpha-1-antitrypsin 1-2**	P22599|A1AT2_MOUSE	59	45975
**Vimentin**	P20152|VIME_MOUSE	71	53688
**Methylmalonate-semialdehyde dehydrogenase [acylating]**	Q9EQ20|MMSA_MOUSE	69	57916
**Tubulin beta-5 chain**	P99024|TBB5_MOUSE	76	49671
**Tubulin beta-2B chain**	Q9CWF2|TBB2B_MOUSE	74	49953
**Tubulin beta-2A chain**	Q7TMM9|TBB2A_MOUSE	73	49907
**Elongation factor Tu**	Q8BFR5|EFTU_MOUSE	68	49508
**Alpha-1-antitrypsin 1-3**	Q00896|A1AT3_MOUSE	57	45823
**Elongation factor 1-alpha 2**	P62631|EF1A2_MOUSE	73	50454
**Elongation factor Tu**	tr|D3YVN7|D3YVN7_MOUSE	68	49538
**Alpha-1-antitrypsin 1-1**	P07758|A1AT1_MOUSE	57	46003
**Selenium-binding protein 2**	Q63836|SBP2_MOUSE	69	52610
**Alpha-1-antitrypsin 1-4**	Q00897|A1AT4_MOUSE	63	45998
**Succinyl-CoA:3-ketoacid coenzyme A transferase 1**	Q9D0K2|SCOT1_MOUSE	71	55989
**EH domain-containing protein 1**	Q9WVK4|EHD1_MOUSE	68	60603
**EH domain-containing protein 2**	Q8BH64|EHD2_MOUSE	70	61175
**Dihydrolipoyllysine-residue acetyltransferase component of pyruvate dehydrogenase complex**	Q8BMF4|ODP2_MOUSE	63	67942
**Glucose-6-phosphate isomerase**	P06745|G6PI_MOUSE	58	62767
**Apoptosis-inducing factor 1**	Q9Z0X1|AIFM1_MOUSE	54	66766
**Cytochrome b-c1 complex subunit 1**	Q9CZ13|QCR1_MOUSE	67	52852
**Protein disulfide-isomerase A3**	P27773|PDIA3_MOUSE	63	56678
**Alpha-1-antitrypsin 1-5**	Q00898|A1AT5_MOUSE	51	45891
**Tripartite motif-containing protein 72**	Q1XH17|TRI72_MOUSE	77	52817
**Tubulin beta-3 chain**	Q9ERD7|TBB3_MOUSE	54	50419
**Rab GDP dissociation inhibitor beta**	Q61598|GDIB_MOUSE	73	50537
**NADH dehydrogenase [ubiquinone] flavoprotein 1**	Q91YT0|NDUV1_MOUSE	77	50834
**Tubulin alpha-1B chain**	P05213|TBA1B_MOUSE	63	50152
**Tubulin alpha-1A chain**	P68369|TBA1A_MOUSE	63	50136
**Dihydrolipoyllysine-residue succinyltransferase component of 2-oxoglutarate dehydrogenase complex**	Q9D2G2|ODO2_MOUSE	59	48995
**Glutamate dehydrogenase 1**	P26443|DHE3_MOUSE	55	61337
**Tubulin beta-4A chain**	Q9D6F9|TBB4A_MOUSE	64	49586
**Propionyl-CoA carboxylase beta chain**	Q99MN9|PCCB_MOUSE	64	58409
**Hydroxysteroid dehydrogenase-like protein 2**	Q2TPA8|HSDL2_MOUSE	68	54208
**Tubulin alpha-1C chain**	P68373|TBA1C_MOUSE	58	49909
**Tubulin alpha-4A chain**	P68368|TBA4A_MOUSE	62	49924
**Acyl-CoA dehydrogenase family member 9**	Q8JZN5|ACAD9_MOUSE	56	68722

**Bifunctional epoxide hydrolase 2**	P34914|HYES_MOUSE	72	62515
**EH domain-containing protein 4**	Q9EQP2|EHD4_MOUSE	55	61481
**Carnitine O-acetyltransferase**	P47934|CACP_MOUSE	40	70840
**Catalase**	P24270|CATA_MOUSE	55	59795
**Lipoamide acyltransferase component of branched-chain alpha-keto acid dehydrogenase complex**	P53395|ODB2_MOUSE	61	53247
**Retinal dehydrogenase 1**	P24549|AL1A1_MOUSE	61	54468
**Elongation factor 1-alpha 1**	P10126|EF1A1_MOUSE	59	50114
**Fibrinogen beta chain**	Q8K0E8|FIBB_MOUSE	56	54753
**Dihydropyrimidinase-related protein 2**	O08553|DPYL2_MOUSE	68	62278
**Methylcrotonoyl-CoA carboxylase beta chain**	Q3ULD5|MCCB_MOUSE	48	61379
**Carboxylesterase 1D**	Q8VCT4|CES1D_MOUSE	58	61788
**Nicotinamide phosphoribosyl transferase**	Q99KQ4|NAMPT_MOUSE	53	55447
**Trifunctional enzyme subunit alpha**	Q8BMS1|ECHA_MOUSE	46	82670
**Serine/threonine-protein phosphatase 2A 65 kDa regulatory subunit A alpha isoform**	Q76MZ3|2AAA_MOUSE	55	65323
**Calsequestrin-2**	O09161|CASQ2_MOUSE	48	48176
**Serine protease inhibitor A3K**	P07759|SPA3K_MOUSE	51	46880
**Tubulin beta-6 chain**	Q922F4|TBB6_MOUSE	46	50090
**Tubulin alpha-3 chain**	P05214|TBA3_MOUSE	48	49960
**Delta-1-pyrroline-5-carboxylate dehydrogenase**	Q8CHT0|AL4A1_MOUSE	43	61841
**NADP-dependent malic enzyme**	Q8BMF3|MAON_MOUSE	55	67098
**Tubulin alpha-8 chain**	Q9JJZ2|TBA8_MOUSE	47	50052
**WD repeat-containing protein 1**	O88342|WDR1_MOUSE	51	66407
**Aspartate--tRNA ligase**	Q922B2|SYDC_MOUSE	50	57147
**Perilipin-1**	Q8CGN5|PLIN1_MOUSE	57	55596
**Amine oxidase [flavin-containing] B**	Q8BW75|AOFB_MOUSE	50	58558
**Fibrinogen gamma chain**	Q8VCM7|FIBG_MOUSE	44	49391
**Sorting and assembly machinery component 50 homolog**	Q8BGH2|SAM50_MOUSE	56	51864
**Protein disulfide-isomerase**	P09103|PDIA1_MOUSE	45	57059
**Pyruvate dehydrogenase protein X component**	Q8BKZ9|ODPX_MOUSE	54	53999
**Adenylyl cyclase-associated protein 1**	P40124|CAP1_MOUSE	56	51565
**Heterogeneous nuclear ribonucleoprotein K**	P61979|HNRPK_MOUSE	46	50976
**Cytosol aminopeptidase**	Q9CPY7|AMPL_MOUSE	53	56141
**Hemopexin OS**	Q91X72|HEMO_MOUSE	44	51318
**Stress-induced-phosphoprotein 1**	Q60864|STIP1_MOUSE	47	62582
**Antithrombin-III**	P32261|ANT3_MOUSE	49	52004
**Calreticulin**	P14211|CALR_MOUSE	46	47995
**UTP--glucose-1-phosphate uridylyltransferase**	Q91ZJ5|UGPA_MOUSE	48	56979
**Malonyl-CoA decarboxylase**	Q99J39|DCMC_MOUSE	58	54736
**Aldehyde dehydrogenase X**	Q9CZS1|AL1B1_MOUSE	47	57553
**Vitamin D-binding protein**	P21614|VTDB_MOUSE	40	53600
**Mitochondrial-processing peptidase subunit alpha**	Q9DC61|MPPA_MOUSE	45	58279
**Rab GDP dissociation inhibitor alpha**	P50396|GDIA_MOUSE	49	50522
**Actin alpha cardiac muscle 1**	P68033|ACTC_MOUSE	40	42019
**ADP/ATP translocase 1**	P48962|ADT1_MOUSE	42	32904
**Histone-lysine N-methyltransferase Smyd1**	P97443|SMYD1_MOUSE	44	56496
**Ig gamma-2B chain C region**	P01867|IGG2B_MOUSE	40	44259

Supplemental Table 6 - Aged heart #3-identities of peptides with a minimum of 40% coverage

Protein Description	Accession	Coverage (%)	Avg. Mass (MW)
**ATP synthase subunit alpha**	Q03265|ATPA_MOUSE	83	59753
**ATP synthase subunit beta**	P56480|ATPB_MOUSE	90	56301
**Serum albumin**	P07724|ALBU_MOUSE	78	68693
**60 kDa heat shock protein**	P63038|CH60_MOUSE	82	60956
**Isocitrate dehydrogenase [NADP]**	P54071|IDHP_MOUSE	77	50906
**Pyruvate kinase**	P52480|KPYM_MOUSE	71	57845
**Beta-enolase**	P21550|ENOB_MOUSE	79	47025
**Trifunctional enzyme subunit beta**	Q99JY0|ECHB_MOUSE	75	51386
**Sarcalumenin**	Q7TQ48|SRCA_MOUSE	43	99184
**Electron transfer flavoprotein-ubiquinone oxidoreductase**	Q921G7|ETFD_MOUSE	59	68091
**Aldehyde dehydrogenase**	P47738|ALDH2_MOUSE	67	56538
**Dihydrolipoyl dehydrogenase**	O08749|DLDH_MOUSE	69	54272
**Tubulin beta-4B chain**	P68372|TBB4B_MOUSE	78	49831
**Phosphoglucomutase-1**	Q9D0F9|PGM1_MOUSE	78	61418
**Alpha-1-antitrypsin 1-3**	Q00896|A1AT3_MOUSE	64	45823
**Tubulin beta-5 chain**	P99024|TBB5_MOUSE	78	49671
**Alpha-1-antitrypsin 1-2**	P22599|A1AT2_MOUSE	61	45975
**Alpha-1-antitrypsin 1-1**	P07758|A1AT1_MOUSE	62	46003
**Alpha enolase**	P17182|ENOA_MOUSE	73	47141
**Fumarate hydratase**	P97807|FUMH_MOUSE	68	54357
**Tubulin beta-2A chain**	Q7TMM9|TBB2A_MOUSE	78	49907
**EH domain-containing protein 2**	Q8BH64|EHD2_MOUSE	78	61175
**Glucose-6-phosphate isomerase**	P06745|G6PI_MOUSE	66	62767
**Propionyl-CoA carboxylase beta chain**	Q99MN9|PCCB_MOUSE	73	58409
**Alpha-1-antitrypsin 1-4**	Q00897|A1AT4_MOUSE	69	45998
**Methylmalonate-semialdehyde dehydrogenase [acylating]**	Q9EQ20|MMSA_MOUSE	68	57916
**Cytochrome b-c1 complex subunit 1**	Q9CZ13|QCR1_MOUSE	60	52852
**Selenium-binding protein 1**	P17563|SBP1_MOUSE	74	52514
**Alpha-1-antitrypsin 1-5**	Q00898|A1AT5_MOUSE	62	45891
**Very long-chain specific acyl-CoA dehydrogenase**	P50544|ACADV_MOUSE	62	70876
**Elongation factor Tu mitochondrial**	Q8BFR5|EFTU_MOUSE	70	49508
**Dihydrolipoyllysine-residue acetyltransferase component of pyruvate dehydrogenase complex**	Q8BMF4|ODP2_MOUSE	55	67942
**Elongation factor Tu**	tr|D3YVN7|D3YVN7_MOUSE	64	49538
**Elongation factor 1-alpha 2**	P62631|EF1A2_MOUSE	68	50454
**Tubulin beta-4A chain**	Q9D6F9|TBB4A_MOUSE	71	49586
**Apoptosis-inducing factor 1**	Q9Z0X1|AIFM1_MOUSE	58	66766
**Rab GDP dissociation inhibitor beta**	Q61598|GDIB_MOUSE	78	50537
**EH domain-containing protein 1**	Q9WVK4|EHD1_MOUSE	78	60603
**Bifunctional epoxide hydrolase 2**	P34914|HYES_MOUSE	66	62515
**Succinyl-CoA:3-ketoacid coenzyme A transferase 1**	Q9D0K2|SCOT1_MOUSE	73	55989
**Tubulin beta-3 chain**	Q9ERD7|TBB3_MOUSE	51	50419
**Tubulin alpha-1A chain**	P68369|TBA1A_MOUSE	71	50136
**Tubulin alpha-1B chain**	P05213|TBA1B_MOUSE	71	50152
**Selenium-binding protein 2**	Q63836|SBP2_MOUSE	66	52610
**Vimentin**	P20152|VIME_MOUSE	65	53688
**Tubulin alpha-1C chain**	P68373|TBA1C_MOUSE	71	49909
**Catalase**	P24270|CATA_MOUSE	49	59795
**Tubulin alpha-4A chain**	P68368|TBA4A_MOUSE	70	49924
**NADH dehydrogenase [ubiquinone] flavoprotein 1**	Q91YT0|NDUV1_MOUSE	60	50834
**Dihydropyrimidinase-related protein 2**	O08553|DPYL2_MOUSE	64	62278
**Tripartite motif-containing protein 72**	Q1XH17|TRI72_MOUSE	69	52817
**EH domain-containing protein 4**	Q9EQP2|EHD4_MOUSE	60	61481
**Dihydrolipoyllysine-residue succinyltransferase component of 2-oxoglutarate dehydrogenase complex**	Q9D2G2|ODO2_MOUSE	59	48995
**Fibrinogen beta chain**	Q8K0E8|FIBB_MOUSE	53	54753

**Protein disulfide-isomerase A3**	P27773|PDIA3_MOUSE	59	56678
**Acyl-CoA dehydrogenase family member 9**	Q8JZN5|ACAD9_MOUSE	53	68722
**Methylcrotonoyl-CoA carboxylase beta chain**	Q3ULD5|MCCB_MOUSE	57	61379
**Carboxylesterase 1D**	Q8VCT4|CES1D_MOUSE	58	61788
**Tubulin alpha-3 chain**	P05214|TBA3_MOUSE	53	49960
**Hydroxysteroid dehydrogenase-like protein 2**	Q2TPA8|HSDL2_MOUSE	64	54208
**Serine/threonine-protein phosphatase 2A 65 kDa regulatory subunit A alpha isoform**	Q76MZ3|2AAA_MOUSE	53	65323
**Aconitate hydratase mitochondrial**	Q99KI0|ACON_MOUSE	42	85464
**Calsequestrin-2**	O09161|CASQ2_MOUSE	46	48176
**Glutamate dehydrogenase 1**	P26443|DHE3_MOUSE	55	61337
**Delta-1-pyrroline-5-carboxylate dehydrogenase**	Q8CHT0|AL4A1_MOUSE	42	61841
**Carnitine O-acetyltransferase**	P47934|CACP_MOUSE	49	70840
**Tubulin alpha-8 chain**	Q9JJZ2|TBA8_MOUSE	54	50052
**Elongation factor 1-alpha 1**	P10126|EF1A1_MOUSE	53	50114
**Protein disulfide-isomerase**	P09103|PDIA1_MOUSE	54	57059
**Nicotinamide phosphoribosyltransferase**	Q99KQ4|NAMPT_MOUSE	54	55447
**Serine protease inhibitor A3K**	P07759|SPA3K_MOUSE	42	46880
**Bifunctional purine biosynthesis protein**	Q9CWJ9|PUR9_MOUSE	52	64217
**Phosphoglucomutase-like protein 5**	Q8BZF8|PGM5_MOUSE	49	62220
**Sorting and assembly machinery component 50**	Q8BGH2|SAM50_MOUSE	55	51864
**Amine oxidase [flavin-containing] B**	Q8BW75|AOFB_MOUSE	62	58558
**T-complex protein 1 subunit beta**	P80314|TCPB_MOUSE	57	57477
**Tubulin beta-6 chain**	Q922F4|TBB6_MOUSE	45	50090
**Lipoamide acyltransferase component of branched-chain alpha-keto acid dehydrogenase complex**	P53395|ODB2_MOUSE	66	53247
**WD repeat-containing protein 1**	O88342|WDR1_MOUSE	52	66407
**Fibrinogen gamma chain**	Q8VCM7|FIBG_MOUSE	46	49391
**UTP--glucose-1-phosphate uridylyltransferase**	Q91ZJ5|UGPA_MOUSE	48	56979
**Citrate synthase**	Q9CZU6|CISY_MOUSE	55	51737
**Histone-lysine N-methyltransferase Smyd1**	P97443|SMYD1_MOUSE	57	56496
**Succinate-semialdehyde dehydrogenase**	Q8BWF0|SSDH_MOUSE	47	55968
**Trifunctional enzyme subunit alpha**	Q8BMS1|ECHA_MOUSE	49	82670
**Mitochondrial-processing peptidase subunit alpha**	Q9DC61|MPPA_MOUSE	47	58279
**Pyruvate dehydrogenase protein X component**	Q8BKZ9|ODPX_MOUSE	57	53999
**NADP-dependent malic enzyme**	Q8BMF3|MAON_MOUSE	45	67098
**Aspartate--tRNA ligase**	Q922B2|SYDC_MOUSE	56	57147
**Heterogeneous nuclear ribonucleoprotein K**	P61979|HNRPK_MOUSE	47	50976
**Retinal dehydrogenase 1**	P24549|AL1A1_MOUSE	51	54468
**UPF0317 protein C14orf159 homolog**	Q8BH86|CN159_MOUSE	40	66366
